# Using partner notification to address curable sexually transmitted infections in a high HIV prevalence context: a qualitative study about partner notification in Botswana

**DOI:** 10.1186/s12889-019-6813-2

**Published:** 2019-05-29

**Authors:** Adriane Wynn, Corrina Moucheraud, Neo Moshashane, Ogechukwu Agatha Offorjebe, Doreen Ramogola-Masire, Jeffrey D. Klausner, Chelsea Morroni

**Affiliations:** 1 GloCal, University of California Global Health Institute, 550 16th Street, 3rd Floor, San Francisco, CA 94158 USA; 2 Division of Infectious Diseases & Global Public Health, Department of Medicine, University of California San Diego, 9500 Gilman Drive, La Jolla, CA 92093 USA; 3 Fielding School of Public Health, Department of Health Policy and Management, University of California Los Angeles, 31-269 CHS, Box 951772, Los Angeles, CA 90095 USA; 4 Botswana-UPenn Partnership, UB Main Campus, Gaborone, Botswana; 5 David Geffen School of Medicine, University of California Los Angeles, 10833 Le Conte Avenue, Los Angeles, CA 90095 USA; 6 Charles R. Drew University of Medicine and Science, 1731 E 120th St, Los Angeles, CA 90059 USA; 7 Faculty of Medicine, University of Botswana, Gaborone, Botswana; 8 Liverpool School of Tropical Medicine, Liverpool, UK; 9 Department of Medicine, University of Botswana, Gaborone, Botswana; 10 Wits Reproductive Health and HIV Institute, University of Witwatersrand, Johannesburg, South Africa; 11 Women’s Health Research Unit, University of Cape Town, Cape Town, South Africa

**Keywords:** Partner notification, Treatment, Sexually transmitted infections, Pregnant women, *Chlamydia trachomatis*, *Neisseria gonorrhoeae*, *Trichomonas vaginalis*, Southern Africa, HIV

## Abstract

**Background:**

Partner notification is an essential component of sexually transmitted infection (STI) management. The process involves identifying exposed sex partner(s), notifying these partner(s) about their exposure to a curable STI, and offering counselling and treatment for the STI as a part of syndromic management or after results from an STI test. When implemented effectively, partner notification services can prevent the index patient from being reinfected with a curable STI from an untreated partner, reduce the community burden of curable STIs, and prevent adverse health outcomes in both the index patient and his or her sex partner(s).

However, partner notification and treatment rates are often low. This study seeks to explore experiences and preferences related to partner notification and treatment for curable STIs among pregnant women receiving care in an antenatal clinic with integrated HIV and curable STI testing. Results are intended to inform efforts to improve partner notification and treatment rates in Southern Africa.

**Methods:**

We conducted qualitative interviews among women diagnosed with *Chlamydia trachomatis* (CT)*, Neisseria gonorrhoeae* (NG), and/or *Trichomonas vaginalis* (TV) infection while seeking antenatal care in Gaborone, Botswana. Semi-structured interviews were used to obtain women’s knowledge about STIs and their experiences and preferences regarding partner notification.

**Results:**

Fifteen women agreed to participate in the study. The majority of women had never heard of CT, NG, or TV infections prior to testing. Thirteen out of 15 participants had notified partners about the STI diagnosis. The majority of notified partners received some treatment; however, partner treatment was often delayed. Most women expressed a preference for accompanying partners to the clinic for treatment. Experiences and preferences did not differ by HIV infection status.

**Conclusions:**

The integration of STI, HIV, and antenatal care services may have contributed to most women’s willingness to notify partners. However, logistical barriers to partner treatment remained. More research is needed to identify effective and appropriate strategies for scaling-up partner notification services in order to improve rates of partners successfully contacted and treated, reduce rates of STI reinfection during pregnancy, and ultimately reduce adverse maternal and infant outcomes attributable to antenatal STIs.

**Electronic supplementary material:**

The online version of this article (10.1186/s12889-019-6813-2) contains supplementary material, which is available to authorized users.

## Background

Partner notification is an essential component of sexually transmitted infection (STI) management, including for HIV and curable infections such as *Chlamydia trachomatis* (CT), *Neisseria gonorrhoeae* (NG), *Trichomonas vaginalis* (TV), and syphilis [1]. The process involves identifying sex partners, notifying them about their exposure, and providing counselling and treatment if appropriate [1, 2]. Partner notification and treatment reduces the likelihood of re-infecting a treated index patient, as per-partnership transmission probabilities are estimated to be high [3], and it may decrease the burden of infection in communities because the partners may be asymptomatic and otherwise unlikely to access the healthcare system for treatment [4–7]. Further, notifying and treating partners for STIs is critical during antenatal care. Even curable infections such as CT, NG, and TV are major causes of morbidity among women and are associated with adverse perinatal and infant outcomes, including preterm birth, and mother-to-child transmission of HIV [7–13].

There are three main approaches to partner notification for curable STIs: (1) *Health professional-oriented methods* where healthcare workers contact the partner(s) of the index patient, inform the partner(s) that they have been exposed to a curable STI, and offer counselling and treatment for the STI (either directly or through referral). (2) *Patient-oriented methods* where the index patient notifies their partners and encourages them to seek medical care or provides treatment directly to their partner(s). (3) *Mixed approaches* involving both the index patient and a healthcare provider. For example, an index patient may be given a deadline to notify their partner(s) and bring them in for treatment, and if the deadline is not met, the provider may contact the partner(s) [14]. Regardless of the strategy utilized, the World Health Organization (WHO) and the Joint United Nations Programme on HIV/AIDS (UNAIDS) recommend that partner notification for HIV and other STIs be conducted on a voluntary basis [15].

Among the above-mentioned strategies, none has been identified as the gold standard and partner notification rates are often low regardless of the strategy used [14, 2]. A systematic review, which examined research related to partner notification strategies in low- and middle-income countries, found that just over half of partners were notified in the 39 included studies [2]. In Botswana, our study setting, recent research found that many pregnant women reported being willing to notify their partners about an STI diagnosis (90%) [16]. This high level of reported willingness could be related to the fact that Botswana has a high antenatal HIV prevalence (33%) and HIV education, opt-out HIV testing, and partner notification messages are routinely provided during antenatal care [17]. However, questions remain about whether willingness translates to actual notification and subsequent partner treatment.

This study seeks to explore experiences and preferences related to partner notification and treatment for curable STIs among pregnant women receiving care in an antenatal clinic with integrated HIV and curable STI testing. This qualitative study aims to inform partner notification services in Southern Africa as well as future research with a deeper understanding of partner notification as a strategy for diagnosing and treating new cases of STIs and HIV.

## Methods

### Study setting

Our study took place in an antenatal clinic in Gaborone, Botswana. In Botswana, antenatal care attendance is high, more than 92% of antenatal care attendees are tested for HIV through a routine, opt-out system, and 33% of pregnant women are infected with HIV [18]. The standard care for curable infections such as CT, NG, and TV, includes identifying and treating infections based on symptoms and clinical signs, called syndromic management. Partner notification is patient-based, and women believed to have an STI are encouraged by health providers to inform their sex partner(s), and are provided with a contact slip to give to partner(s) that details the symptom being treated (e.g. abnormal vaginal discharge), the treatment (e.g. azithromycin), and the date and location of treatment [19]. This study was nested within a CT, NG, and TV infection testing and treatment intervention (main study) that was integrated into an antenatal care clinic using the GeneXpert® System (Cepheid, Sunnyvale, CA, USA). The main study offered the standard care for partner services but differed from syndromic management of STIs by making an etiologic diagnosis of STIs and treating based on this diagnosis (not signs and symptoms).

### Participant selection and recruitment

Between August 2016–February 2017, a sample of women who tested positive for CT, NG, or TV at the Princess Marina Hospital antenatal clinic in Gaborone, Botswana were recruited by phone for participation in the qualitative study by a female, Setswana and English-speaking researcher. In the main study, per the standard of care in Botswana, after testing positive for an STI, women received a partner contact slip with information about the STI they were diagnosed with, the treatment provided, and space for a partner’s healthcare provider to sign to confirm that the partner was treated. Further, women were counselled to notify partners, encourage partners to get treatment, and avoid sex for seven days after treatment [20]. Women were advised to return for a test of cure after four weeks. In order to understand a variety of perspectives on partner notification, for this qualitative study we aimed to recruit participants diagnosed with different STIs (e.g. CT, NG, or TV), with and without HIV coinfection, and who did and did not tell their partners about their STI diagnosis. All women were pregnant (< 35 weeks of gestation), at least 18 years of age, and receiving care at Princess Marina at the time of the STI diagnosis. Those who agreed to participate in this qualitative study were scheduled for an in-person, 30 min to 1-h interview in a private office on the University of Botswana campus or a location of their choosing. Participants were given a 30 pula (~ 3 USD) reimbursement for their transportation. Prior to the interview, participants provided written informed consent.

The interviews were guided by an outline of open-ended questions and probes that explored the following domains: participant’s general well-being, sexual relationship status and history, HIV infection status, experiences with STI testing, partner notification experiences, barriers and facilitators to notifying their partners, partner reaction and treatment outcomes, and preferences related to partner notification. The guide was pilot tested on two participants and revised to improve understanding. The study was also temporarily paused after seven interviews, and transcripts were reviewed to ensure the guide was understood and facilitated collection of detailed and candid information. Each transcript also included a fieldnotes section where the interviewer described the setting, rapport with the participant and any other thoughts relevant to the interview.

### Data collection

One-on-one interviews were conducted in Setswana or English by a female researcher who had a University degree, training in qualitative methods, and extensive experience in interviewing on women’s reproductive health issues. Referrals to health organizations and community-based services for depression, domestic violence, or health concerns were provided as needed.

Interviews were digitally recorded, transcribed verbatim, and translated to English. As a quality control check, after seven transcriptions were completed, every other transcript was selected (3 total) and translated independently by another member of the study team. Discrepancies between the two translations were identified and discussed. However, only minor changes were made.

### Data analysis

To develop a codebook, four transcripts were selected at random and codes were extracted by two study team members based on themes included in the interview guide while also allowing for new codes to emerge inductively from the unique data [21]. The codes were then compared and aggregated into a master codebook with codes and definitions. All transcripts were read and coded using this codebook in Microsoft Word and Excel. Coded transcripts were assessed for frequently used codes and patterns associated with women’s experiences related to partner notification of an STI. Illustrative quotations were extracted for dominant themes. Divergent or minority views were also noted. Narratives and themes were compared between participants to understand similarities and differences. Reporting was based on the consolidated criteria for reporting qualitative research (COREQ) [22].

## Results

### Participant characteristics

A convenience sample of 22 women were contacted and 15 were enrolled in the study. Of the seven who were not enrolled, four initially agreed and then continually postponed, two people moved away from Gaborone, and in one instance, the study interviewer declined to stay for an interview due to safety concerns. Characteristics collected at the time of STI testing, including demographic and relationships characteristics, HIV infection status, STI-related symptoms, and self-reported partner notification outcomes, were compared between those who refused and enrolled. There did not appear to be any appreciable differences between those who participated and those who did not participate in terms of age, marital status, education level, or HIV infection status. Further, participants in the qualitative interviews were similar in terms of age, HIV-status, and STI-related symptoms to those diagnosed with a curable STI in the main study population. Among the seven who did not enroll, five reported that they told their partners about the STI diagnosis and one reported that her partner was not treated (results not shown).

Table 1 describes the age, marital status, highest education level, HIV infection status, self-reported STI symptoms, partner notification outcomes, and test of cure results for the study sample. The mean age was 29 years, all were unmarried, and 6 (40%) had achieved a tertiary level of education. The prevalence of HIV infection was 40% (6) and almost a third (4) reported having abnormal vaginal discharge at time of STI testing. No women reported having multiple sex partners in the year prior to the STI diagnosis. Thirteen women reported notifying their partners about their positive STI diagnosis, and seven reported that their partners were treated, four said that their partners were not treated, and four said that they weren’t sure if their partners were treated. Three women in the sample retested positive for an STI at test of cure, which took place four weeks after initial testing.

**Table 1:** Characteristics of women participants in the sexually transmitted infection partner notification study at Princess Marina Hospital in Gaborone, Botswana (*N* = 15)

	Study Sample N (%)
Age in years, Mean (range)	29 (21–35)
Unmarried	15 (100%)
Education	
Junior secondary or less	6 (40%)
Senior secondary	3 (20%)
Tertiary	6 (40%)
HIV-infected	6 (40%)
Self-reported STI-related symptoms at time of STI testing^a^	7 (47%)
Vaginal discharge	4 (57%)
Painful urination	2 (29%)
Lower abdominal pain	3 (43%)
Told partner about STI diagnosis	13 (87%)
Partner treated	
Yes	7 (47%)
No (includes those who were not notified)	4 (27%)
Unsure	4 (27%)
STI cured in index participant at follow-up	12 (80%)

Note: ^a^Some women reported more than one symptom. The denominators for vaginal discharge, painful urination, and lower abdominal pain is the number of women with any symptomsPercentages may not add up to 100 due to rounding

Table 2 describes each participant’s STI diagnosis, HIV infection and partner status, self-reported partner notification and partner treatment outcomes, and test of cure outcome. Most women in our qualitative sample were infected with CT-only (9/15) and still in a relationship with the baby’s father (10/15). The two women, who did not tell partners about the STI diagnosis, were no longer in a relationship with the baby’s father. Both dissolved their relationships prior to learning of the STI diagnosis. Four women reported that their partners were not treated, including two who were not treated despite being notified. Four women were unsure if their partners were treated because they did not have proof (e.g. by accompanying him to the clinic or being provided with a contact slip signed by a doctor/nurse). Among the three women who retested positive for CT at follow-up, one was unsure if her partner was treated. The time between initial STI test and interview ranged from 5 to 20 months.

**Table 2:** Characteristics of women included in the qualitative interview sample

Participant ID	STI	HIV Infection Status	Partner status^a^	Partner notified	Partner treated	STI cured^b^
1	TV	Infected	No partner	No	No	Yes
2	TV	Uninfected	New partner	No	No	Yes
3	CT	Infected	Baby’s father	Yes	No	Yes
4	CT	Infected	No partner	Yes	No	Yes
5	NG	Infected	Baby’s father	Yes	Unsure	Yes
6	CT	Uninfected	Baby’s father	Yes	Unsure	No
7	TV	Uninfected	Baby’s father	Yes	Unsure	Yes
8	CT	Uninfected	No partner	Yes	Unsure	Yes
9	CT	Uninfected	Baby’s father	Yes	Yes	Yes
10	CT	Uninfected	Baby’s father	Yes	Yes	No
11	CT	Uninfected	Baby’s father	Yes	Yes	No
12	CT	Infected	Baby’s father	Yes	Yes	Yes
13	TV	Uninfected	Baby’s father	Yes	Yes	Yes
14	TV	Uninfected	Baby’s father	Yes	Yes	Yes
15	CT	Infected	No partner	Yes	Yes	Yes

Note: ^a^ Partner status at time of interview. ^b^ Participant retested at four weeks for cure

### Knowledge about CT, NG, or TV infections

Ten of the 15 women had never heard of CT, NG, or TV infections prior to testing, including all six of the women living with HIV. Three women said that they had heard about the infections, and two reported only hearing about gonorrhea.

### Reasons for testing for CT, NG, or TV infections

When asked about their motivation for STI testing during their pregnancy, seven women mentioned that they wanted to test in order to know if they had infections and expressed an understanding that they could be infected without knowing.

*Because most of the time, we will be living with infections, but not knowing, so I wanted to see.* (Participant 3, age 27) ***

*I felt that it was important to do that [test] because you never know, maybe some things stay in you, you can have them without symptoms you see.* (Participant 4, age 33)

Two women said that they tested to protect the baby from infections. “*… for the sake of my baby, so that’s why I wanted to.”* (Participant 2, age 28*)* Two women said that it was an opportunity to test that they would not normally have. Two women reported that they had symptoms they thought may be a result of an STI or had been previously treated for an STI and wanted to see if it they were still infected.

*I had challenges of, for two months I used to get itchy down there and I’d ask myself why, you see.* (Participant 6, age 31)

While only one woman mentioned partner infidelity as a reason for testing, seven women reported that their partners were likely having sex with other women and one woman said “*… he is all over the place. There’s no one that doesn’t know him.”* (Participant 12, age 25) Two women ended relationships with their partners because they impregnated other women. Alcohol use was discussed by five women as a contributor to infidelity. *“Yes, when I ask him, he says he was drunk and didn’t know what he was doing.”* (Participant 1, age 24).

### Women’s reaction to STI diagnosis

Four of the 15 women reported that they were “okay” with their positive STI results or “accepted” them and did not choose to elaborate further upon probing. Only a few reported that they were very surprised to be infected and the remainder expressed relief or an appreciation for being able to receive treatment for an infection.

*Now, when I was told, I just accepted that, yes, maybe they’ll help me. I just really wanted help.* (Participant 11, age 21) ***

*That’s why I accepted because even if I had received wrong results [testing positive], I knew I would be helped, and the baby.* (Participant 9, age 28)

### Partner notification experiences

Among the 13 women who told their partners about the STI results, three had recently separated from their partners and the remainder were still with the partner who they had been with for one year or longer at the time of notification. In notifying their partners, most women told them in person, without much delay from time of diagnosis and were straightforward in sharing the news. All but a few women reported using the contact slip to help inform their partners about the STI results.

*I told him that “Mr. I was told that we have STI’s.” … And again I showed him the clinic card, because you had marked it somewhere.* (Participant 1, age 24) ***

*Yes. I didn’t go around in circles, I got in and said, I was in [the clinic] and there were people testing for sexually transmitted diseases so I also tested, but came back positive. The disease is called Chlamydia now you can read these papers and see what kind of disease it is.* (Participant 5, age 35)

The only barrier to notification identified by women who told their partners was distance, when the partner lived in another city, which caused delays in notification. Those who waited did not want to share this information over the phone. *“Hey, [this news is] sensitive and can’t be said over the phone.*” (Participant 7, age 33) One person shared the results through an image of her medical record on Whatsapp.

Reasons for telling partners were generally multi-faceted and included wanting to protect the partner’s health, prevent reinfection, and not wanting to keep a secret from the partner.

*Because we are together, we sleep together. So obviously, what I have I must share with him. So that if he also needs help he may get it.* (Participant 4, age 33)

One woman said that counselling provided in the clinic encouraged her to ensure that her partner was treated.

*The advice that [clinic staff] gave me is the one that gave me that courage to tell them. [They] told me it's safe to get treated for that and my boyfriend to get treated…Because there will be no point of me getting treated and him not.* (Participant 11, age 21)

The two women who did not notify their partners were no longer in a relationship with the baby’s father at the time that they received the STI results. One woman did not know how to get in contact with the partner and one was reluctant to communicate after the breakup.

Among the six women living with HIV, two were no longer in relationships and hadn’t notified previous partners about their HIV status. Four women living with HIV had notified their current partners previously about their HIV status. One woman explained that she had notified all of her sex partners about her HIV status.

*Before I can get into any relationship, whether you judge me or what, I have to tell you.* (Participant 5, age 35)

Despite having increased familiarity with partner notification due to their HIV status, women living with HIV infection did not report different experiences with partner notification compared to those who were uninfected. All, but one woman with HIV notified their partners about the curable STI. One woman did not notify because she was no longer in a relationship.

### Partner’s reactions to STI diagnosis

Among participants who notified their partners, the majority reported that their partners reacted well to being informed about the STI results. Six women reported that their partners said “it’s okay” or “it will be fine” after being told about the diagnosis. Two partners were reported to be scared, one for the baby’s safety and the other about getting an injection. One partner made a joke.

*“ … Then they found that I have this infection.” (Starts laughing) Then he just said “it’s loving sex, that’s the only problem.”* (Participant 7, age 33)

One participant reported that her partner reacted in anger and she had sex with him to calm him down.

*I said “I went to [the clinic] for a checkup and then I checked myself [tested].” Now he is shouting at me for checking myself … “What did you check for?” saying “you like testing yourself for so many things!” This and that. “So you think I sleep around with girls, am I sick?” Right then we had sex again because he was shouting right … Yes, I was calming him down.* (Participant 12, age 25)

Some partners asked questions and participants didn’t have enough information to answer.

*I said, “Don’t bother me with too many questions I don’t want questions, you will ask for yourself. There’s a lot of time, they give you time to ask.”* (Participant 8, age 31)

*He just asked what it was. I said, “I don’t know I’m asking you, let’s go.”* (Participant 1, age 24)

### Partner treatment experiences

Women encouraged partners to seek treatment in a variety of ways. One woman said that she would not have sex with him until he received treatment, *“We are not going to have sex until you’re tested”*, another woman said,*“ if you want a child again let’s go check again for STI’s”* (Participant 5, age 35), and one woman said that she’s protecting him by telling him to get treated for the STI, *“dude, do you see how much I protect you?”* (Participant 2, age 28) A few partners did not seek treatment until contacted by clinic staff, at the participant’s request, to encourage treatment uptake.

*So they [partners] come with us. Because when we tell them they refuse. You see that I asked him and then after [clinic staff] called that’s when he came.* (Participant 12, age 25)

The two un-notified partners were assumed to be untreated. Among the two notified partners that were not treated, one participant reported that his work schedule was a barrier to receiving care at a clinic. Another woman, who was no longer together with the baby’s father reported, *“it was just laziness,”* (Participant 10, age 32) that prevented her ex-partner from seeking care. Several women reported that their partners may not have been treated if the treatment was injection. Several women reported having problems getting the partner treated when they didn’t have the contact slip. One partner was confused about what to say when he arrived at the clinic without a contact slip.

*He told me that, when he gets to the hospital what should he say. And I told him “no when you get to the hospital, there’s no evidence that I can give you, when you get to the hospital you tell them my partner was tested and she was found with STI’s.”* (Participant 5, age 35)

Many women explained that it’s difficult to get male sex partners to access health care even for HIV testing. For example, five of 15 women in our sample, including three women living with HIV, did not know their partners’ HIV infection status, and reported that their partners were likely “*testing through me*.” Several women mentioned that their partner was unwilling to get tested because he could check his status when she got tested.

*Yes, because when I said go and test, I tested myself, he asked me “are you ok” and I said “I’m fine” then he said “yes that means I’m fine.” Do you see the issue?* (Participant 12, age 25) ***

*He is very difficult when it comes to testing. When I go and test and then show him he believes he is also ok.* (Participant 5, age 35)

Among treated partners, half of the women accompanied them to the clinic. When partners went to the clinic on their own, some participants had doubts that they were treated.

*I’ll just have to believe I can’t dispute it. [Interviewer: He hasn’t shown you his card or anything?] No, he hasn’t shown me.* (Participant 9, age 28)

While most women were cured when tested approximately 4 weeks after STI diagnosis and treatment, three women retested positive for CT at the first test of cure. One of these women did not notify her partner after the first diagnosis and had sex without a condom. Thereafter, she notified him, he was treated, and her second test of cure was negative. Similarly, the remaining two women’s partners were treated only after the first test of cure was positive, and in both cases clinic staff called to encourage the partners to seek treatment.

### Preferences for notifying partners in the future

Participants were asked questions about how they might want to notify a partner in the future and different options were described to them. When asked, in general, how they would prefer to notify partners in the future, most women preferred to tell their partner themselves in person and generally thought the way they told him went well. Only the woman whose partner was angry with her upon notification preferred to have a healthcare provider notify.

*Me as a woman, I can tell him. If it’s a problem and he can’t understand, that’s when I can take him to you [clinic staff] so you explain what we are talking about.* (Participant 6, age 31)

We also asked how women preferred that partners get treatment, and described possible options, which included: bringing treatment home to partners (e.g. women would bring information and treatment home for their partners to take prior to him being examined by a healthcare provider), have partners go to the clinic alone (with probing questions on whether a contact slip was sufficient or if a provider should call), or accompany partners to the clinic. Most participants said that they would like to accompany their partners to the clinic for treatment because many said that otherwise he may not go.

*But if you give me the paper [contact slip] I’m going to need to go with him because if I don’t he won’t do it [get treated].* (Participant 10, age 32) ***

*I would need me to come with him. If you call him and say he should come, he is going to agree and not come. It needs me to say let’s go, they called you. (Participant 15, age 25)*

No women preferred to bring treatment home to their partners. Two women explained that they would not want to bring treatment home because the partner would have many questions or would refuse the treatment.

*Ah, it wasn’t going to be good. He was going to refuse … He was going to ask himself what pills I was giving to him that he hasn’t been told about.* (Participant 1, age 24)

## Discussion

We assessed pregnant women’s experiences and preferences associated with partner notification of an STI diagnosed during antenatal care in a setting with a high HIV prevalence. Among our sample of 15 women, most had never heard of CT, NG, or TV infections before testing. All but two notified their partners and among those who notified, distance (e.g. when the partner lived in another city) was described as a barrier. Most women used the contact slip to notify their partners and encourage them to get treatment. Women who didn’t notify their partners were no longer in relationships. Just under half of women reported that their partners were definitely treated, and the remainder said their partners were not treated or they weren’t certain that their partners were treated. Women who tested positive for an STI at the test of cure reported that partners delayed receiving treatment. Several women needed to have a healthcare provider call to encourage the partner to get treatment. Reported barriers to treatment were the partner’s work schedule and a fear of injections. Many women reported concerns that their partners were having sex with other women. In terms of future preferences, all but one woman reported that they would want to tell their partner about an STI diagnosis themselves. Most women would want to accompany their partners to the clinic for treatment and none would prefer to take medication home to the partner. Although women living with HIV may have had more experience in terms communicating with partners about STIs compared to uninfected women, their experiences and preferences related to CT, NG, and TV infection notification and treatment did not differ.

We found that pregnant women were willing to notify their partners about an STI, however, this willingness did not always result in partners being treated [2]. Motivations from previous qualitative research in Southern Africa were similar to our findings in that women were motivated to tell partners because they thought sex partners were the source of infection and needed treatment, or to protect a child from infection [23]. However, barriers to telling partners also included: partners lived far away, embarrassment, and fearing losing support or intimate partner violence (IPV) [23, 24]. Although none of our participants reported IPV, careful monitoring is still needed, as the prevalence of IPV has been found to be high in previous studies in Botswana [25, 26]. Settings with high levels of IPV may consider an IPV screen to identify women in violent relationships who may not be able to participate in partner notification programs if her safety cannot be assured.

Even when partners were treated, several received delayed treatment, which puts pregnant women at risk for reinfection, and reduces the effectiveness of antenatal STI testing and treatment. A recent modelling study found that reducing partner treatment from fourteen to one or two days substantially reduced the risk of CT/NG reinfection of an index patient [3]. Further, delays in partner treatment have been previously identified as a concern in Botswana. A 2013 study assessed contact slips of partners treated for an STI from approximately 285 health facilities in Botswana to identify any delays between index patient and partner treatment. This study found that, among partners who reported for treatment, 22.1% were treated a week or more after the index patient [27].

In order to improve and expedite partner treatment rates, several new strategies have been proposed and implemented in other settings, such as the United States [28]. One strategy is expedited partner therapy, where an index patient brings medication home to partner(s) prior to the partner’s evaluation by a healthcare provider [28]. Several randomized controlled trials have shown that expedited partner therapy can reduce reinfection rates compared to simple patient referral (patient tells sex partners they need to be treated) [29]. One study involving men and women in the United States, who were randomized to expedited partner therapy or simple partner referral, found that 13% of index patients in the simple referral group had a persistent or recurrent gonococcal or chlamydial infection compared to only 10% in the expedited partner therapy group [30].

However, given that many women in our study expressed some concerns about expedited partner therapy, decisions about future services should also consider strategies that ease the burden on women to ensure that partners are treated, such as provider-based notification or enhanced patient notification activities, such as providing additional information about STIs for the index patient and partner(s). In fact, a systematic review found that enhanced patient referrals, (including home sampling kits for partners, educational information for patients to give to partners, and disease-specific websites) were just as effective at preventing reinfection as expedited partner treatment [29]. Additionally, a study in the UK compared time to partner treatment between routine patient-referral and two accelerated partner treatment (APT) methods, including: 1) APT Hotline where partner(s) received assessment and consultation by healthcare providers over the phone and either collected treatment at clinic reception or had it delivered by the index patient. 2) APT Pharmacy where the sex partner(s) attended a pharmacy for consultation and treatment [31]. This study found that the median number of days between index patient diagnosis and sex partner treatment was shorter for the APT Hotline (1 day, range 10–14 days, *p* = 0.05) and APT Pharmacy (2 days, range 0–6 days, *p* = 0.09) compared to routine partner notification (3 days, range 0–17) [31]. Such strategies could help address the concern expressed by many of our participants that they would not be able to answer all of their partners’ questions.

Participants in our study were unwilling or unable to notify previous partners about an STI diagnosis, which is a finding similar to previous research in Southern Africa [23, 24]. While women are not at risk of reinfection from ex-partners, not notifying a likely STI case may represent a missed opportunity to reduce infections in the community. Studies have estimated that 70–80% of partners of index cases with NG are infected and 60–70% of partners of index cases with CT are infected [32, 33].

In circumstances where women are unable or unwilling to notify their former partners themselves, it may be possible for electronic communication technologies to play a role, such as SMS, or web-based notification. Although little research has taken place in sub-Saharan Africa, there is growing research on the acceptability and utilization of these technologies for STI notification [34]. Further, many participants expressed concerns that their partners may have other casual sex partners who could potentially also be reached through electronic communication if their partners are unwilling to tell them in person. Previous studies found that referral strategies requiring less interaction, were preferred for notifying ex-partners or casual partners [3].

## Limitations

The study has some limitations. First, our sample was small and was derived from a single clinical site that serves women from Gaborone and southern Botswana. As previously reported, the sample of women tested for CT, NG, and TV infections from which women were recruited for this study had characteristics similar to the population of pregnant women in Botswana in terms of age, and marital and HIV status [17, 35]. Second, participants who participated in the qualitative study may be different than women who did not with respect to partner notification. For example, our sample may include those more willing to discuss partner notification because they were more successful in notifying their partners. Third, response bias is almost always a limitation when participants are asked sensitive questions. However, it was encouraging to read in the transcript fieldnotes sections that our trained interviewer interpreted most women’s responses to be honest and open. Fourth, our study included only pregnant women, and findings are not generalizable to non-pregnant women or men diagnosed with an STI in Botswana. Previous research has found that pregnant women may be more likely be in long term relationships and to notify their partners due to concerns about the baby compared to non-pregnant women [36].

Similarly, no study participants reported having multiple sex partners in the year prior to diagnosis. This finding is not surprising as a previous study found that only 5% of pregnant women in Gaborone had two or more partners in the last 12 months [4]. Another, more recent article, found in a nationally representative survey that, on average, 6% of (nonpregnant) women reported two or more sexual partners in the past month [37]. While we were unable to assess experiences and preferences among women with multiple partners, this issue is critical to consider when implementing new partner notification strategies. Recent research has highlighted the importance of tailoring partner notification services to different partner types (e.g. steady committed, new relationship, occasional, one-off partner), which may enhance effectiveness and cost-effectiveness [38].

Finally, it is important to note that this study took place within a larger STI testing study that deviated from the standard care in Botswana, syndromic management, where curable STIs are treated based on signs and symptoms [19]. Syndromic management is not sensitive, missing many infections, and not specific, potentially overtreating pregnant women [4]. As such, when index infections are missed, so are those of partners. Another flaw is that women may be encouraged to disclose an STI that they don’t have, potentially exposing them unnecessarily to negative partner reactions, such as IPV [2].

In Botswana, partner notification services for HIV are similar to curable STIs. Women who test positive for HIV are encouraged by health providers to inform their sex partners, and guidelines call for providers to offer additional counselling to women who are reluctant or fearful to disclose [39]. Further, providers are allowed to inform a woman’s partner only in her presence and upon her request [39]. Our study highlights the disparity between men and women in terms of accessing health services, which has been found in HIV research [40]. This disparity not only puts men’s health at risk as they are less likely receive HIV testing and treatment [40], but it also places a burden on women to protect their partner(s)’ health in order to protect their own. As such, the enhanced partner notification and provider-based notification or case finding recommended for curable STIs may also facilitate notification for HIV. Further, as management of curable STIs and HIV are continuing to integrate into antenatal care in Botswana, it may be possible to harmonize partner notification services to streamline the process and increase rates of notification and linkage to care for multiple infections.

## Conclusions

In conclusion, the aim of our study was to gain a more detailed understanding about the experiences and preferences of pregnant women related to notifying partners about an STI in a setting with a high antenatal HIV prevalence. The integration of STI, HIV, and antenatal care services may have contributed to most women’s willingness to notify partners. However, logistical barriers to partner treatment remained. In order to improve rates of partner notification and treatment, reduce rates of re-infection during pregnancy, and subsequently reduce adverse maternal and infant outcomes due to antenatal STIs; more research is needed to identify effective and appropriate strategies for partner treatment.

A French translation of this article has been included as Additional file 1.

A Portuguese translation of the abstract has been included as Additional file 2.

## Additional files

Additional file 1: Translation of this article into French. (PDF 303 kb)

Additional file 2: Translation of the abstract of this article into Portuguese. (PDF 376 kb)

## Abbreviations

APT: Accelerated partner treatment
COREQ: Consolidated criteria for reporting qualitative research
CT: *Chlamydia trachomatis*
HIV: Human immunodeficiency virus
IPV: Intimate partner violence
NG: *Neisseria gonorrhoeae*
STI: Sexually transmitted infections
TV: *Trichomonas vaginalis*
UNAIDS: Joint United Nations Programme on HIV/AIDS
USD: United States dollar
WHO: World Health Organization
