# Supplementary material for: Using partner notification to address curable sexually transmitted infections in a high HIV prevalence context: a qualitative study about partner notification in Botswana
Source: BMC Public Health. 2019 May 29;19(Suppl 1):606. doi: 10.1186/s12889-019-6813-2 (PMC6538557; doi:10.1186/s12889-019-6813-2)
Supplement: Supplementary file 1 — Translation of this article into French. (PDF 303 kb) [file 12889_2019_6813_MOESM1_ESM.pdf]

Prévenir les partenaires pour combattre les infections sexuellement transmissibles curables dans un contexte de forte prévalence du VIH : Étude qualitative sur l'annonce aux partenaires au Botswana

Adriane Wynn[1,2*], Corrina Moucheraud[3], Neo Moshashane[4], Ogechukwu Agatha Offorjebe[5,6], Doreen Ramogola-Masire[7], Jeffrey D Klausner[8], Chelsea Morroni[9, 10, 11, 12, 13]

[1]GloCal, University of California Global Health Institute, 550 16th Street, 3rd Floor, San Francisco, CA 94158, USA,
[2] Division of Infectious Diseases & Global Public Health, Department of Medicine, University of California, San Diego, 9500 Gilman Drive, La Jolla, CA 92093, USA, Email : awynn@ucsd.edu ,
[3]Department of Health Policy and Management, University of California, Los Angeles, 31-269 CHS Box 951772, Los Angeles, CA 90095, USA, Email : cmoucheraud@g.ucla.edu,
[4] Botswana-UPenn Partnership, UB Main Campus, Gaborone, Botswana, Email : Moshashanen@bup.org.bw
[5]David Geffen School of Medicine, University of California, Los Angeles, 10833 Le Conte Avenue, Los Angeles, CA 90095, USA, Email : OOfforjebe@mednet.ucla.edu
[6]Charles R. Drew University of Medicine and Science, 1731 E 120th St, Los Angeles, CA 90059
[7]Faculty of Medicine, University of Botswana, Gaborone, Botswana, Email : doreen.masire@gmail.com
[8] David Geffen School of Medicine, University of California, Los Angeles, 10833 LE Conte Avenue, Los Angeles, CA 90095, USA, Email : JDKlausner@mednet.ucla.edu
[9] Botswana-UPenn Partnership, UB Main Campus, Gaborone, Botswana
[10] Liverpool School of Tropical Medicine, UK
[11] Department of Medicine, University of Botswana, Gaborone, Botswana
[12] Wits Reproductive Health and HIV Institute, University of Witwatersrand, Johannesburg, South Africa
[13] Women's Health Research Unit, University of Cape Town, South Africa, E-mail : chelseaamorroni@gmail.com

Résumé

Contexte : L'annonce aux partenaires est une composante essentielle de la gestion des infections sexuellement transmissibles (IST). Ce processus implique d'identifier le ou les partenaire(s) sexuel(s) exposé(s), d'informer ce(s) partenaire(s) de leur exposition à une IST curable et de proposer un accompagnement et un traitement pour l'IST dans le cadre de la prise en charge syndromique ou après avoir obtenu les résultats du dépistage. Lorsqu'ils sont utilisés efficacement, les services d'annonce aux partenaires peuvent empêcher le patient de référence d'être réinfecté par une IST curable par un partenaire non soigné, réduire le risque d'IST curables au sein de la communauté et éviter les problèmes de santé à la fois chez le patient de référence et chez ses partenaires sexuels.

Cependant, les taux d'annonce et de traitement des partenaires sont souvent bas. Cette étude vise à analyser les expériences et les préférences en matière d'annonce et de traitement des partenaires pour les IST curables des femmes enceintes prises en charge dans un service de soins prénatals pouvant effectuer un dépistage du VIH et des IST curables. Les résultats ont

pour but de fournir des informations sur les actions à mettre en place pour améliorer les taux d'annonce et de traitement des partenaires en Afrique australe.

Méthodes : Nous avons mené des entretiens qualitatifs auprès de femmes à qui l'on avait diagnostiqué une infection par *Chlamydia trachomatis* (CT), *Neisseria gonorrhoeae* (NG) et/ou *Trichomonas vaginalis* (TV) au cours d'une consultation de soins prénatals à Gaborone, au Botswana. Des entretiens semi-structurés ont été réalisés pour recueillir les connaissances des femmes sur les IST ainsi que leurs expériences et préférences en matière d'annonce aux partenaires.

Résultats : Quinze femmes ont accepté de participer à l'étude. La plupart des femmes n'avaient jamais entendu parler des infections par CT, NG ou TV avant le dépistage. Treize de 15 participantes avaient informé leurs partenaires du diagnostic d'IST. La majorité des partenaires informés a reçu un traitement. Cependant, le traitement des partenaires a souvent été tardif. La plupart des femmes ont indiqué qu'elles préféraient accompagner leurs partenaires à la clinique pour le traitement. Les expériences et les préférences n'ont pas varié en fonction du statut d'infection par le VIH.

Conclusions : Il est possible que le recours à des services de traitement des IST, du VIH et de soins prénatals ait contribué à la volonté de la plupart des femmes d'informer leurs partenaires. Cependant, des obstacles logistiques au traitement des partenaires subsistent. Des recherches supplémentaires sont nécessaires pour identifier les stratégies efficaces et adaptées afin d'améliorer les services d'annonce aux partenaires dans le but de faire augmenter les taux de partenaires contactés et traités avec succès, baisser le taux de réinfection par des IST au cours de la grossesse et enfin d'éviter les problèmes de santé de la mère et du nouveau-né attribuables à des IST contractées pendant la grossesse.

Mots clés : annonce aux partenaires, traitement, infections sexuellement transmissibles, femmes enceintes, *Chlamydia trachomatis, Neisseria gonorrhoeae, Trichomonas vaginalis*, Afrique australe, VIH

Contexte

L'annonce aux partenaires est une composante essentielle de la gestion des infections sexuellement transmissibles (IST), y compris les infections par le VIH et les infections curables comme *Chlamydia trachomatis* (CT), *Neisseria gonorrhoeae* (NG), *Trichomonas vaginalis* (TV) et la syphilis.[1] Ce processus implique d'identifier les partenaires sexuels, de les informer de leur exposition et de proposer un accompagnement et un traitement si nécessaire.[1, 2] L'information et le traitement des partenaires réduisent le risque de réinfection d'un patient de référence traité, étant donné que les probabilités de transmission par le partenaire sont élevées,[3] et cela peut réduire le risque d'infection au sein de la communauté puisque les partenaires peuvent être asymptomatiques ou bien peu susceptibles d'avoir accès au système de santé pour être traités.[4-7] De plus, il est essentiel d'informer et de traiter les partenaires pour leurs IST dans le cadre des soins prénatals. Même les infections curables comme CT, NG et TV sont des causes majeures de morbidité chez les femmes et sont associées à des problèmes de santé au cours de la grossesse et chez le nouveau-né, y compris des naissances prématurées et une transmission mère-enfant du VIH.[7-13]

Il existe trois approches principales en termes d'annonce au partenaire dans le cas d'une IST curable : (1) **Les méthodes reposant sur les professionnels de santé**, dans lesquelles les prestataires de soins contactent le ou les partenaire(s) du patient de référence, le ou les informe(nt) de l'exposition à une IST curable et proposent un accompagnement et un

traitement de l'IST (directement ou en les orientant vers le service adapté). (2) **Les méthodes reposant sur les patients**, dans lesquelles le patient de référence informe ses partenaires et les incite à consulter un médecin ou leur fournit le traitement directement. (3) **Les approches mixtes** impliquant à la fois le patient de référence et un prestataire de soins. Par exemple, on peut donner au patient une date limite pour informer ses partenaires et les amener consulter, et si la date limite est dépassée, le prestataire de soins peut contacter les partenaires.[14] Quelle que soit la méthode utilisée, l'Organisation mondiale de la Santé (OMS) et le programme commun des Nations Unies sur le VIH/SIDA (ONUSIDA) recommandent que l'annonce au partenaire pour le VIH et les autres IST soit effectuée de manière volontaire.[15]

Aucune des stratégies mentionnées ci-dessus n'a été identifiée comme la meilleure à suivre, et les taux d'annonce aux partenaires sont souvent bas quelle que soit la méthode utilisée.[14, 16] Une analyse systématique, qui a étudié les recherches portant sur les stratégies d'annonce aux partenaires dans les pays à revenus faibles et moyens, a établi qu'un peu plus de la moitié des partenaires ont été informés dans les 39 études analysées.[16] Au Botswana, le cadre de notre étude, des études récentes ont indiqué que de nombreuses femmes enceintes ont signalé vouloir annoncer à leurs partenaires un diagnostic d'IST (90%).[17] Ce taux élevé de bonnes intentions pourrait être lié au fait que le Botswana possède une forte prévalence prénatale du VIH (20,5%), une bonne sensibilisation au VIH, des dépistages du VIH et que les messages d'annonce au partenaire sont délivrés de manière classique lors des soins prénatals.[18] Cependant, des questions subsistent sur le fait que cette bonne volonté se traduise par une réelle annonce et un traitement subséquent du partenaire.

Cette étude vise à analyser les expériences et les préférences en matière d'annonce et de traitement des partenaires pour les IST curables des femmes enceintes prises en charge dans un service de soins prénatals pouvant effectuer un dépistage du VIH et des IST curables. Cette étude qualitative a pour but de renseigner les services d'annonce aux partenaires en Afrique australe ainsi que de fournir aux futures études une compréhension plus profonde de l'annonce au partenaire en tant que stratégie de diagnostic et de traitement de nouveaux cas d'IST et de VIH.

Méthodes

Configuration de l'étude

Notre étude s'est déroulée dans un centre de soins prénatals à Gaborone au Botswana. Au Botswana, la fréquentation des services de soins prénatals est élevée, plus de 92% des patientes de ces services sont testées pour le VIH dans le cadre d'un système de dépistage de routine et 33% des femmes enceintes sont séropositives.[19] Le parcours de soins classique pour les infections curables comme CT, NG et TV consiste à identifier et à traiter les infections en se basant sur les symptômes et les signes cliniques (prise en charge syndromique). L'annonce aux partenaires repose sur les patients, et les femmes qu'on pense infectées par une IST sont encouragées par les prestataires de soins à l'annoncer à leurs partenaires sexuels. On leur fournit une fiche de contact à donner à leurs partenaires qui détaille les symptômes traités (par ex. écoulement vaginal anormal), le traitement (par ex. azithromycine) ainsi que la date et le lieu du traitement.[20] Cette étude a été imbriquée dans une recherche sur le dépistage et le traitement des infections par CT, NG et TV (étude principale) qui a été réalisée dans un centre de soins prénatals en utilisant le système GeneXpert® (Cepheid, Sunnyvale, Californie, États-

Unis). L'étude principale proposait un parcours de soins classique pour les partenaires mais différait de la prise en charge syndromique des IST en réalisant un diagnostic étiologique des IST et en effectuant le traitement sur la base de ce diagnostic et non sur la base des signes et des symptômes.

Sélection et recrutement des participantes

Entre août 2016 et février 2017, un échantillon de femmes dont le dépistage, réalisé au centre de soins prénatals du Princess Marina Hospital de Gaborone au Botswana, avait été positif pour CT, NG ou TV ont été recrutées par téléphone pour participer à une étude qualitative par une chercheuse parlant le tswana et l'anglais. Dans l'étude principale, dans le cadre du parcours de soins classique au Botswana, après un dépistage positif pour une IST, les femmes ont reçu une fiche de contact pour leur partenaire avec des informations sur l'IST diagnostiquée, le traitement apporté et un espace destiné à la signature du prestataire de soins du partenaire pour confirmer que celui-ci avait été traité. Ensuite, il a été conseillé aux femmes d'informer leurs partenaires, de les encourager à suivre un traitement et d'éviter les rapports sexuels pendant sept jours après le traitement.[21] Il leur a également été conseillé de revenir faire un test pour vérifier l'efficacité du traitement au bout de quatre semaines. Dans le but de comprendre plusieurs points de vue sur l'annonce au partenaire, nous avons tenté de recruter pour cette étude qualitative des participantes auxquelles on avait diagnostiqué diverses IST (par ex. CT, NG ou TV), avec ou sans co-infection par le VIH, et qui avaient annoncé ou non à leurs partenaires le diagnostic d'IST. Toutes les femmes étaient enceintes (moins de 35 semaines de grossesse), âgées d'au moins 18 ans et suivies au Princess Marina au moment du diagnostic d'IST. Pour celles qui ont accepté de participer à cette étude qualitative, un entretien en face à

face d'une durée de 30 min à 1h a été programmé dans un local privé sur le campus de l'université du Botswana ou sur le lieu de leur choix. Les participantes ont été défrayées à hauteur de 30 pula (environ 3 USD) pour le transport. Les participantes ont donné leur consentement par écrit avant l'entretien.

Les entretiens étaient guidés par une suite de questions ouvertes portant sur les domaines suivants : le bien-être général des participantes, leur statut et leurs antécédents en matière de relations sexuelles, leur statut VIH, leurs expériences de dépistage des IST, leurs expériences de l'annonce à leurs partenaires, les éléments bloquant ou favorisant l'annonce aux partenaires, la réaction des partenaires et le fait qu'ils aient suivi le traitement ou non et leurs préférences concernant l'annonce aux partenaires. Le guide a d'abord été testé sur deux participantes puis révisé pour améliorer la compréhension. L'étude a également été temporairement suspendue au bout de sept entretiens et les transcriptions ont été relues pour s'assurer que le guide était bien compris et qu'il facilitait la collecte d'informations détaillées et sincères. Chaque transcription comprenait également une partie de notes contextuelles dans laquelle l'enquêtrice décrivait le cadre de l'entretien, le rapport avec la participante et toute autre réflexion pertinente au sujet de l'entretien.

Collecte des données

Des entretiens en face à face ont été menés en tswana ou en anglais par une chercheuse diplômée de l'enseignement supérieur, formée aux méthodes qualitatives et possédant une solide expérience des entretiens avec des femmes sur les questions de santé reproductive. Les participantes ont pu si nécessaire être orientées vers des organismes de santé

et des services communautaires dans les cas de dépression, de violences conjugales ou de questions de santé.

Les entretiens ont été enregistrés en format numérique, transcrits textuellement et traduits en anglais. En guise de contrôle qualité, toutes les sept transcriptions, chaque transcription supplémentaire était sélectionnée (3 au total) et traduite de manière indépendante par un autre membre de l'équipe de recherche. Les différences entre les deux traductions étaient identifiées et faisaient l'objet d'un débat. Seuls quelques changements mineurs ont été apportés.

Analyse des données

Pour développer un livre-code, quatre transcriptions ont été sélectionnées au hasard et les codes ont été extraits par deux membres de l'équipe de l'étude sur la base des thèmes inclus dans le guide d'entretien, tout en autorisant l'émergence inductive de nouveaux thèmes à partir de données particulières.[22] Les codes ont ensuite été comparés et agrégés dans un livre-code principal contenant les codes et les définitions. Toutes les transcriptions ont été lues et codées en utilisant ce livre-code dans Microsoft Word et Excel. Les transcriptions codées ont été évaluées à la recherche de codes et de schémas fréquemment utilisés et associés aux expériences des femmes en matière d'annonce aux partenaires d'une infection par une IST. Des citations d'illustrations ont été extraites pour les thèmes les plus souvent abordés. Les avis divergents ou minoritaires ont également été repérés. Les récits et les thèmes ont été comparés entre les participantes pour comprendre les ressemblances et les différences. L'analyse a été effectuée en utilisant les critères consolidés pour l'analyse d'une recherche qualitative (COREQ).[23]

Résultats

Caractéristiques des participantes

Un échantillon de commodité de 22 femmes a été contacté et 15 d'entre elles ont été enrôlées dans l'étude. Sur les sept qui n'ont pas été enrôlées, quatre ont accepté avant de se rétracter, deux personnes ont déménagé et quitté Gaborone et dans le dernier cas, l'enquêtrice a refusé l'entretien pour des raisons de sécurité. Les données recueillies au moment du dépistage de l'IST, y compris les données démographiques, le type de relation, le statut d'infection par le VIH, les symptômes liés à l'IST et les résultats de l'annonce au partenaire donnés par les participantes, ont été comparées entre celles qui ont accepté l'entretien et celles qui l'ont refusé. Aucune différence notable n'a été soulignée entre les participantes et les femmes n'ayant pas pris part à l'étude en termes d'âge, de statut matrimonial, de niveau d'études ou de statut d'infection par le VIH. De plus, les participantes aux entretiens qualitatifs étaient semblables en termes d'âge, de statut VIH et de symptômes liés à l'IST aux femmes à qui l'on avait diagnostiqué une IST curable parmi la population de l'étude principale. Parmi les sept femmes qui n'ont pas participé à l'étude, cinq ont indiqué avoir informé leurs partenaires du diagnostic d'IST et une a déclaré que son partenaire n'avait pas été traité (résultats non présentés).

Le Tableau 1 reprend l'âge, le statut matrimonial, le plus haut niveau d'études atteint, le statut d'infection par le VIH, les symptômes de l'IST mentionnés par la participante, les résultats de l'annonce au partenaire et les résultats du test de guérison de l'échantillon étudié. La moyenne d'âge était de 29 ans, aucune femme n'était mariée et 6 d'entre elles (40%) étaient diplômées de l'enseignement supérieur. La prévalence de l'infection par le VIH était de 40% (6)

et quasiment un tiers (4) ont indiqué constater des écoulements vaginaux anormaux au moment du dépistage de l'IST. Aucune femme n'a indiqué avoir eu plusieurs partenaires sexuels au cours de l'année précédant le diagnostic d'IST. Treize femmes ont indiqué avoir informé leur partenaire du diagnostic d'IST. Sept femmes ont déclaré que leur partenaire avait été traité, quatre ont déclaré que leur partenaire n'avait pas été traité et quatre ont déclaré ne pas être sûres que leur partenaire avait été traité. Trois femmes de l'échantillon ont obtenu un résultat positif lors du test de guérison de l'IST, qui a eu lieu quatre semaines après le dépistage initial.

Le Tableau 2 reprend le diagnostic d'IST, le statut d'infection par le VIH, le statut du partenaire, l'annonce au partenaire, le fait que le partenaire ait été traité ou non et les résultats du test de guérison de chaque participante. La plupart des femmes de notre échantillon qualitatif étaient infectées uniquement par CT (9/15) et vivaient toujours une relation avec le père du bébé (10/15). Les deux femmes qui n'ont pas informé leurs partenaires du diagnostic d'IST n'étaient plus en couple avec le père de leur bébé. Les relations de couple de ces deux femmes ont pris fin avant le diagnostic d'IST. Quatre femmes ont indiqué que leurs partenaires n'avaient pas été traités, y compris deux qui n'ont pas été traités bien qu'ils aient été informés. Quatre femmes n'étaient pas sûres que leurs partenaires avaient été traités car elles n'avaient aucune preuve (elles ne les avaient par exemple pas accompagnés à la clinique ou n'avaient pas récupéré de reçu signé d'un médecin ou d'une infirmière). Parmi les trois femmes dont le test de suivi est revenu positif pour CT, une n'était pas sûre que son partenaire ait été traité. Le temps écoulé entre le dépistage initial de l'IST et l'entretien était de 5 à 20 mois.

Connaissance des infections par CT, NG ou TV

Dix des 15 femmes n'avaient jamais entendu parler des infections par CT, NG ou TV avant le dépistage, y compris les six femmes séropositives. Trois femmes ont indiqué avoir entendu parler de ces infections, et deux ont déclaré avoir entendu parler uniquement de la blennorragie.

Motifs du dépistage des infections par CT, NG ou TV

Lorsqu'elles ont été interrogées sur les motifs d'un dépistage d'IST pendant leur grossesse, sept femmes ont indiqué qu'elles voulaient être dépistées pour savoir si elles avaient des infections et ont montré qu'elles comprenaient qu'elles pouvaient être infectées sans le savoir.

> *Parce que la plupart du temps, on vit avec les infections sans même le savoir, alors je voulais voir.* (Participante 3, 27 ans)
> ***
> *Pour moi, c'était important de faire ça [le test] parce qu'on ne sait jamais, peut-être que certaines choses restent dans le corps, on peut les avoir sans ressentir de symptômes, vous voyez.* (Participante 4, 33 ans)

Deux femmes ont déclaré s'être fait dépister pour protéger le bébé des infections. *« ... pour la santé de mon bébé, c'est pour ça que je voulais le faire. »* (Participante 2, 28 ans) Deux femmes ont expliqué que c'était une opportunité de se faire dépister dont elles ne disposent pas habituellement. Deux femmes ont mentionné qu'elles avaient des symptômes qui selon elles pouvaient être dus à une IST ou qu'elles avaient déjà été traitées pour une IST et qu'elles voulaient voir si elles étaient toujours infectées.

> *Je me posais des questions, pendant deux mois, j'ai ressenti des démangeaisons dans cette zone, alors je me demandais pourquoi, vous voyez.* (Participante 6, 31 ans)

Bien qu'une seule femme ait évoqué l'infidélité de son partenaire comme motif de dépistage, sept femmes ont indiqué qu'il était possible que leur partenaire ait des rapports

sexuels avec d'autres femmes, et l'une des participantes a dit *« Il traîne partout. Tout le monde le connaît. »* (Participante 12, 25 ans) Deux femmes ont mis fin à leur relation avec leur partenaire parce qu'une autre femme était tombée enceinte de lui. La consommation d'alcool a été évoquée par cinq femmes comme un facteur favorisant l'infidélité. *« Oui, quand je lui ai posé la question, il m'a dit qu'il était ivre et qu'il ne savait pas ce qu'il faisait. »* (Participante 1, 24 ans)

Réaction des femmes au diagnostic d'IST

Quatre femmes sur 15 ont déclaré ne pas avoir eu de problème avec les résultats positifs du dépistage ou les avoir acceptés et n'ont pas souhaité en parler davantage lorsqu'elles ont été interrogées à ce sujet. Seules quelques-unes ont indiqué avoir été très surprises d'être infectées et les participantes restantes ont été soulagées ou reconnaissantes de pouvoir recevoir un traitement pour l'infection.

> *En fait, quand on me l'a dit, j'ai juste accepté le fait que, peut-être, ils allaient pouvoir m'aider. Je voulais juste vraiment qu'on m'aide.* (Participante 11, 21 ans)
> ***
> *C'est pour ça que j'ai accepté, parce que je savais que même si je recevais de mauvais résultats [test positif], on allait m'aider, et mon bébé aussi.* (Participante 9, 28 ans)

Expériences d'annonce au partenaire

Parmi les 13 femmes ayant annoncé les résultats du dépistage d'IST à leur partenaire, trois s'étaient récemment séparées de leurs partenaires et les participantes restantes vivaient encore en couple avec le partenaire qu'elles avaient depuis un an ou plus au moment de l'annonce. Pour informer leurs partenaires, la plupart des femmes ont préféré une annonce de vive voix, sans attendre très longtemps après le diagnostic, et ont été directes. Presque toutes

les femmes ont indiqué avoir utilisé la fiche de contact pour faciliter l'annonce des résultats du dépistage d'IST à leur partenaire.

> *Je lui ai dit « On m'a dit que nous avons des IST. » Et je lui ai montré à nouveau la carte de clinique, parce que cela avait été noté dessus quelque part.* (Participante 1, 24 ans)
> *****
> *Oui. Je n'ai pas tourné autour du pot, j'ai pris la carte et j'ai dit que j'étais allée [à la clinique], que des gens proposaient un dépistage des infections sexuellement transmissibles et que j'en avais passé un qui était revenu positif. La maladie s'appelle Chlamydia, maintenant tu peux lire ces documents et voir de quel genre de maladie il s'agit.* (Participante 5, 35 ans)

Le seul obstacle à l'information identifié par les femmes qui ont annoncé le résultat à leur partenaire était la distance, lorsque le partenaire vivait dans une autre ville, ce qui a retardé l'annonce. Celles qui ont attendu ne souhaitaient pas annoncer la nouvelle par téléphone. *« Quand même, [c'est un sujet] sensible qu'on ne peut pas évoquer par téléphone. »* (Participante 7, 33 ans) Une personne a partagé les résultats en envoyant une photo de son dépistage par Whatsapp.

Les motifs de l'annonce au partenaire étaient en général multiples. Il s'agissait notamment d'une volonté de protéger la santé du partenaire, d'éviter la réinfection et de ne pas vouloir cacher quelque chose au partenaire.

> *Parce qu'on est ensemble, on couche ensemble. Alors forcément, si j'ai quelque chose, il l'a aussi. Donc s'il a besoin d'aide lui aussi, il pourra en obtenir.* (Participante 4, 33 ans)

Une femme a indiqué que l'accompagnement réalisé à la clinique l'a encouragée à s'assurer que son partenaire soit traité.

> *C'est le conseil [du personnel de la clinique] qui m'a donné le courage le lui dire. [Ils] m'ont dit que le traitement était sans risque et que mon copain devait être traité ... Parce que ça ne servait à rien de me traiter moi et de ne pas le traiter lui.* (Participante 11, 21 ans)

Les deux femmes qui n'ont pas informé leurs partenaires ne vivaient plus une relation avec le père de leur bébé au moment du diagnostic de l'IST. Une femme ne savait pas comment contacter son partenaire et l'autre était réticente à l'idée de communiquer après la rupture.

Parmi les six femmes séropositives, deux n'étaient plus en couple et n'avaient pas annoncé leur séropositivité à leurs partenaires précédents. Quatre femmes séropositives avaient informé leurs partenaires actuels de leur séropositivité auparavant. Une femme a expliqué avoir annoncé sa séropositivité à tous ses partenaires sexuels.

> *Avant de commencer n'importe quelle relation, et peu importe si l'on me juge, je le dis.* (Participante 5, 35 ans)

Même si elles étaient davantage familiarisées avec l'annonce au partenaire du fait de leur séropositivité, les femmes séropositives n'ont pas évoqué d'expériences différentes en termes d'annonce au partenaire par rapport aux participantes séronégatives. Toutes les femmes séropositives sauf une ont annoncé leur IST curable à leur partenaire. L'une d'entre elles ne l'a pas fait car elle n'était plus en couple.

Réaction des partenaires au diagnostic d'IST

Parmi les participantes ayant informé leurs partenaires, la plupart ont indiqué que ceux-ci avaient bien réagi au fait d'être informés des résultats du dépistage d'IST. Six femmes ont déclaré que leur partenaire avait répondu « pas de problème » ou « ça va aller » après l'annonce du diagnostic. Deux partenaires ont été inquiets, l'un pour la sécurité du bébé et l'autre à l'idée de recevoir une injection. L'un des partenaires a plaisanté.

> *« Et ils ont découvert que j'avais cette infection. » (La participante se met à rire) Il a juste répondu « On aime le sexe, c'est ça le seul problème. »* (Participante 7, 33 ans).

Une participante a indiqué que son partenaire s'est mis en colère et qu'elle a eu un rapport sexuel avec lui pour le calmer.

> *Je lui ai dit « Je suis allée [à la clinique] pour un suivi et je me suis fait dépister. » Il s'est mis à me crier dessus pour le dépistage ... « Pourquoi est-ce que tu t'es fait dépister ? », disait-il « Tu aimes te faire dépister pour tellement de choses ! » Ce genre de choses. « Alors tu penses que je couche avec d'autres filles, que je suis malade ? » Ensuite, nous avons eu un rapport sexuel encore une fois, parce qu'il criait tellement ... Oui, j'essayais de le calmer.* (Participante 12, 25 ans)

Certains partenaires ont posé des questions et les participantes n'avaient pas assez d'informations pour répondre.

> *Je lui ai dit « Ne m'embête pas en me posant trop de questions, je ne veux pas de questions, tu iras demander toi-même. Tu peux prendre ton temps, ils te laissent le temps de poser des questions. »* (Participante 8, 31 ans).

> *Il a simplement demandé ce que c'était. Je lui ai dit « Je ne sais pas, je te demande juste qu'on y aille. »* (Participante 1, 24 ans)

Expériences de traitement du partenaire

Les femmes ont encouragé leurs partenaires à suivre le traitement de diverses manières. Une participante a déclaré qu'elle n'aurait plus de rapport sexuel avec lui dans qu'il n'aurait pas suivi le traitement (*« Nous n'aurons pas de rapport sexuel avant que tu te sois fait dépister »*), une autre femme a dit *« Si tu veux un autre enfant, allons nous faire dépister à nouveau pour les IST »* (Participante 5, 35 ans) et une femme a affirmé qu'elle le protégeait en l'incitant à aller se faire traiter pour l'IST (*« Tu ne vois pas à quel point je te protège ? »*) (Participante 2, 28 ans). Quelques partenaires n'ont pas cherché à se faire traiter jusqu'à ce que la clinique les contacte, sur demande des participantes, pour les encourager dans cette voie.

> *Ils [les partenaires] sont enfin venus avec nous. Parce que quand nous leur demandions, ils refusaient. Vous voyez, je lui ai demandé, puis ils [le personnel de la clinique] ont appelé et là il s'est décidé à y aller.* (Participante 12, 25 ans)

On suppose que les deux partenaires qui n'ont pas été informés n'ont pas été traités. Parmi les deux partenaires mis au courant qui n'ont pas été traités, l'une des participantes a indiqué que l'emploi du temps professionnel de son partenaire compliquait l'accès aux soins à la clinique. Une autre femme, qui n'était plus en couple avec le père de son bébé, a indiqué que *« c'était uniquement la paresse »* (Participante 10, 32 ans) qui avait empêché son ex-compagnon de suivre le traitement. Plusieurs femmes ont mentionné que leurs partenaires ne s'étaient peut-être pas fait soigner si le traitement était sous forme d'injection. Plusieurs femmes ont indiqué avoir eu des problèmes à inciter leur partenaire à se faire traiter lorsqu'elles n'avaient pas la fiche de contact. Un partenaire ne savait pas exactement quoi dire lorsqu'il est arrivé à la clinique sans fiche de contact.

> *Il m'a demandé ce qu'il était censé dire lorsqu'il arriverait à l'hôpital. Et je lui ai dit « Je n'ai pas de papier à te donner pour ta consultation à l'hôpital, quand tu arriveras là-bas, dis-leur que ta partenaire a été dépistée et qu'elle a découvert qu'elle avait une IST. »* (Participante 5, 35 ans)

Beaucoup de femmes ont expliqué qu'il était difficile d'inciter leurs partenaires masculins à se faire soigner, y compris pour le dépistage du VIH. Par exemple, cinq des 15 femmes de notre échantillon, dont trois femmes séropositives, ne connaissaient pas le statut VIH de leur partenaire et ont indiqué que leurs partenaires *« comptaient sur elles pour se faire dépister ».* Plusieurs femmes ont mentionné que leur partenaire ne souhaitait pas se faire dépister car ils pouvaient vérifier leur statut lorsque les participantes passaient le dépistage.

> *Oui, parce que quand je lui ai dit d'aller se faire dépister, que j'y étais allée moi-même, il m'a dit « tout va bien de ton côté ? » et je lui ai répondu que oui, alors il*

> *a dit « ça veut dire que tout va bien de mon côté. » Vous voyez le problème ?*
> (Participante 12, 25 ans)
> *****
> *Il est très pénible quand il s'agit de dépistage. Quand je vais me faire dépister et que je lui montre les résultats, il pense que tout va bien pour lui aussi.*
> (Participante 5, 35 ans)

Parmi les partenaires traités, la moitié ont été accompagnés à la clinique par les participantes. Lorsque les partenaires sont allés seuls à la clinique, certaines participantes ont douté qu'ils se soient fait soigner.

> *Je dois juste le croire, je ne peux pas remettre ça en question. [Enquêtrice : Il ne vous a pas montré sa carte ou quoi que ce soit ?] Non, il ne m'a rien montré.*
> (Participante 9, 28 ans)

Alors que la plupart des femmes étaient guéries lorsqu'elles se sont fait dépister à nouveau, environ 4 semaines après le diagnostic et le traitement pour l'IST, trois femmes étaient à nouveau positives pour CT lors du premier test de guérison. L'une de ses femmes n'avait pas informé son partenaire après le premier diagnostic et avait eu un rapport sexuel non protégé. Par la suite, elle le lui a annoncé, il s'est fait soigner et son second test de guérison était négatif. De même, les partenaires des deux autres femmes n'ont suivi le traitement qu'une fois que le premier test de guérison est revenu positif, et dans les deux cas le personnel de la clinique a appelé les partenaires pour les encourager à se faire soigner.

Préférences pour l'annonce aux partenaires à l'avenir

Les participantes ont été interrogées sur la manière dont elles pourraient vouloir informer un partenaire à l'avenir et plusieurs options leur ont été proposées. En général, lorsqu'on les a questionnées à ce sujet, la plupart des femmes ont déclaré qu'elles préféraient l'annoncer à leur partenaire elles-mêmes, en personne, et elles pensaient globalement que la

manière dont elles l'avaient fait était appropriée. Seule la femme dont le partenaire s'est mis en colère suite à son annonce a déclaré préférer que ce soit un prestataire de soins qui l'annonce.

> *Moi, en tant que femme, je peux le lui dire. Si c'est un problème et qu'il ne comprend pas, à ce moment-là, je peux vous l'amener [au personnel de la clinique] pour que vous lui expliquiez de quoi il s'agit.* (Participante 6, 31 ans)

Nous avons également demandé aux femmes de quelle manière elles préféraient que leurs partenaires suivent le traitement, avec les options suivantes : apporter le traitement à leur partenaire chez elles (par ex. la femme rapporterait les informations et le traitement chez elle et son partenaire le prendrait avant d'être examiné par un prestataire de soins), envoyer leur partenaire consulter seul à la clinique (avec des questions plus approfondies pour savoir si une fiche de contact était suffisante ou s'il était préférable qu'un prestataire de soins appelle) ou accompagner leur partenaire à la clinique. La plupart des participantes ont déclaré qu'elles préfèreraient accompagner leurs partenaires à la clinique pour le traitement car beaucoup pensent que sans ça, leur partenaire ne s'y rendrait pas.

> *Mais si vous me donnez le document [fiche de contact], je dois y aller avec lui, sinon il n'ira pas [suivre le traitement].* (Participante 10, 32 ans)
> *****
> *Je devrai y aller avec lui. Si vous l'appelez pour lui dire qu'il devrait venir, il acquiescera mais il ne viendra pas. C'est moi qui devrai lui dire « ils t'ont appelé, allons-y ». (Participante 15, 25 ans)*

Aucune femme n'a préféré apporter le traitement à son partenaire chez elle. Deux femmes ont expliqué qu'elles n'apporteraient pas le traitement chez elles car leur partenaire poserait beaucoup de questions ou refuserait de le prendre.

> *Ah, ça n'aurait pas marché. Il aurait refusé ... Il se serait demandé quels sont ces comprimés que je lui aurais donnés sans qu'il soit au courant.* (Participante 1, 24 ans)

DISCUSSION

Nous avons évalué les expériences et les préférences de femmes enceintes en matière d'annonce au partenaire d'une IST diagnostiquée dans le cadre des soins prénatals dans un contexte de forte prévalence du VIH. Parmi notre échantillon de 15 femmes, la plupart des femmes n'avaient jamais entendu parler des infections par CT, NG ou TV avant le dépistage. Toutes sauf deux ont informé leurs partenaires et parmi celles qui l'ont annoncé, la distance (c'est-à-dire le fait que le partenaire vive dans une autre ville) a été évoquée comme un obstacle. La plupart des femmes ont utilisé la fiche de contact pour informer leur partenaire et l'encourager à suivre le traitement. Les femmes qui n'ont pas informé leurs partenaires n'étaient plus en couple. Un peu moins de la moitié des femmes ont déclaré que leur partenaire avait été traité, et les autres ont déclaré que leur partenaire n'avait pas été traité ou qu'elles n'étaient pas sûres que leur partenaire ait été traité. Les femmes dont le test de guérison de l'IST est revenu positif ont indiqué que leurs partenaires avaient repoussé le moment de suivre le traitement. Plusieurs femmes ont eu besoin qu'un prestataire de soins appelle pour encourager leur partenaire à suivre le traitement. Les obstacles à l'accès au traitement qui ont été évoqués étaient l'emploi du temps professionnel du partenaire et la peur des piqûres. Beaucoup de femmes ont dit s'inquiéter que leur partenaire ait des rapports sexuels avec une autre femme. En ce qui concerne les préférences pour une future annonce, toutes les femmes sauf une ont indiqué qu'elles aimeraient annoncer elles-mêmes le diagnostic d'IST à leur partenaire. La plupart des participantes souhaiterait accompagner leur partenaire à la clinique pour le traitement et aucune ne préfèrerait rapporter le traitement à leur partenaire chez elles. Même si les femmes séropositives ont peut-être plus d'expérience en matière de communication avec leurs partenaires au sujet des IST que les femmes séronégatives, leurs

expériences et leurs préférences concernant l'annonce et le traitement d'une infection par CT, NG ou TV étaient similaires.

Nous avons découvert que les femmes enceintes souhaitaient informer leur partenaire de leur IST, mais que cette intention ne débouchait pas toujours sur un traitement du partenaire.[16] Les motifs évoqués lors de précédentes études qualitatives menées en Afrique australe étaient similaires à nos résultats : les femmes souhaitaient informer leur partenaire car elles pensaient que celui-ci était à l'origine de l'infection et devait être soigné, ou bien elles voulaient protéger leur bébé de l'infection.[24] Cependant, elles se heurtaient à des obstacles : l'éloignement géographique du partenaire, la grossesse et la peur de perdre le soutien du partenaire ou que celui-ci se montre violent lors des rapports sexuels (VS).[24, 25] Bien qu'aucune de nos participantes n'ait mentionné de VS, un suivi attentif est toujours nécessaire, car de précédentes études ont révélé que la prévalence des VS est élevée au Botswana.[26, 27] Il faudrait envisager une détection des VS dans les environnements présentant un fort taux de VS afin d'identifier les femmes aux conjoints violents qui pourraient ne pas être en mesure de participer aux programmes d'annonce au partenaire si leur sécurité ne peut pas être assurée.

Même dans les cas où les partenaires ont suivi le traitement, plusieurs d'entre eux ont retardé ce moment, ce qui a entraîné un risque de réinfection pour la femme enceinte et réduit l'efficacité du dépistage et du traitement prénatal. Une étude de modélisation récente a démontré que le fait de raccourcir le délai de traitement du partenaire de quatorze à un ou deux jours pouvait réduire de manière substantielle le risque de réinfection par CT/NG pour la patiente de référence.[3] De plus, le fait que le partenaire repousse le moment du traitement a

déjà été identifié comme une source d'inquiétude au Botswana. En 2013, une étude a analysé les fiches de contact de partenaires traités pour une IST dans environ 285 établissements de santé au Botwana pour noter le délai entre le traitement du patient de référence et celui du partenaire. L'étude a découvert que, parmi les partenaires qui se sont présentés pour un traitement, 22,1% ont été traités une semaine ou plus après le patient de référence.[28]

Pour améliorer et accélérer le traitement des partenaires, plusieurs nouvelles stratégies ont été proposées et mises en place dans d'autres endroits, les États-Unis par exemple.[29] L'une d'entre elles est appelée « traitement accéléré du partenaire ». Cela consiste à faire apporter le traitement au partenaire par le patient de référence, chez eux, avant que le partenaire ne soit examiné par un prestataire de soins.[29]Plusieurs essais contrôlés randomisés ont montré que le traitement accéléré du partenaire peut réduire les taux de réinfection par rapport à une simple orientation du patient (le patient informe ses partenaires sexuels qu'ils doivent se faire soigner).[30] Une étude réalisée aux États-Unis, dans laquelle des hommes et des femmes étaient randomisés entre le traitement accéléré du partenaire ou la simple orientation du patient, a démontré que 13% des patients de référence du groupe de la simple orientation souffraient d'une infection persistante ou récurrente par CT ou NG, alors qu'ils n'étaient que 10% dans le groupe du traitement accéléré du partenaire.[31]

Cependant, étant donné que de nombreuses femmes de notre étude ont exprimé des inquiétudes quant au traitement accéléré du partenaire, les décisions sur les futurs services devraient également étudier des stratégies qui évitent aux femmes de devoir s'assurer que leur partenaire a bien été traité, par exemple une annonce réalisé par un prestataire de soins ou des actions renforcées d'annonce aux patients, par exemple en fournissant davantage

d'informations sur les IST au patient de référence et à ses partenaires. En effet, une analyse systématique a établi qu'un renforcement de l'orientation des patients (incluant des kits de dépistage à réaliser chez soi pour le partenaire, des informations éducatives à destination du partenaire et des sites Internet dédiés à chaque maladie) était tout aussi efficace pour éviter la réinfection que le traitement accéléré du partenaire.[30] De plus, une étude réalisée au Royaume-Uni a comparé le temps écoulé avant le traitement du partenaire entre une orientation du patient classique et deux méthodes de traitement accéléré du patient (TAP). Dans le cas de la première méthode, le partenaire pouvait contacter une hotline dédiée au TAP où il était évalué et examiné par téléphone par un prestataire de soins avant de récupérer son traitement à l'accueil de l'hôpital ou de se le faire apporter par le patient de référence. Pour la deuxième méthode, le partenaire se rendait dans une pharmacie dédiée au TAP pour une consultation et un traitement.[32] Cette étude a établi que le nombre médian de jour entre le diagnostic du patient de référence et le traitement du partenaire était plus court dans le cas de la hotline (1 jour, étendue 10-14 jours, p = 0,05) et de la pharmacie (2 jours, étendue 0-6 jours, p = 0,09) que dans le cas de l'annonce classique au partenaire (3 jours, étendue 0-17).[32] Ce type de stratégies pourrait aider à éliminer l'inquiétude exprimée par beaucoup de nos participantes de ne pas pouvoir répondre à toutes les questions de leur partenaire.

Les participantes de notre étude ne souhaitaient pas ou ne pouvaient pas informer leurs partenaires précédents du diagnostic d'IST, un résultat similaire à ceux de précédentes études menées en Afrique Australe.[24, 25] Même si les femmes ne courent pas de risque d'être réinfectées par un ancien partenaire, ne pas signaler un cas possible d'IST peut représenter une opportunité manquée de réduire les infections au sein de la communauté. Selon les recherches,

environ 70 à 80% des partenaires de patients de référence infectés par NG sont aussi infectés et 60 à 70% des partenaires de patients de référence infectés par CT sont aussi infectés.[33, 34]

Dans les cas où les femmes ne peuvent pas ou ne souhaitent pas informer leurs partenaires elles-mêmes, on pourrait peut-être utiliser les technologies de communication comme un SMS ou une notification sur Internet. Même si peu d'études ont été menées en Afrique sub-saharienne, de plus en plus de recherches portent sur l'acceptation et l'utilisation de ces technologies pour l'annonce d'une IST.[35] De plus, de nombreuses participantes étaient inquiètes à l'idée que leur partenaire puisse avoir d'autres partenaires sexuelles occasionnelles, qui pourraient éventuellement être contactées par voie électronique si leurs partenaires ne souhaitent pas les informer de vive voix. De précédentes études ont démontré que les stratégies d'orientation demandant moins d'interactions sont privilégiées pour prévenir les anciens partenaires ou les partenaires occasionnels.[3]

LIMITES

L'étude a quelques limites. Premièrement, notre échantillon était réduit et issu d'une seule clinique dans laquelle consultent les femmes de Gaborone et du sud du Botswana. Comme expliqué précédemment, l'échantillon de femmes dépistées pour des infections par CT, NG et TV au sein duquel les participantes à cette étude ont été recrutées présentait des caractéristiques similaires à celles de la population des femmes enceintes du Botswana en termes d'âge, de statut matrimonial et d'infection par le VIH.[18, 36] Deuxièmement, les participantes à l'étude qualitative ont pu être différentes des femmes qui n'y ont pas pris part en ce qui concerne l'annonce au partenaire. Il est par exemple possible que notre échantillon ait inclus des femmes plus enclines à évoquer l'annonce au partenaire parce que l'annonce à

leur propre partenaire s'est bien déroulée. Troisièmement, le biais méthodologique constitue presque toujours une limite lorsque l'on pose des questions délicates aux participantes. Cependant, il a été encourageant de lire dans les notes contextuelles de la transcription que notre enquêtrice aguerrie a considéré la plupart des réponses des femmes comme étant honnêtes et ouvertes. Quatrièmement, notre étude n'a été menée que sur des femmes enceintes, et les résultats ne sont pas généralisables aux femmes non enceintes ou aux hommes à qui l'on diagnostique une IST au Botswana. De précédentes études ont montré que les femmes enceintes sont davantage susceptibles de s'engager dans des relations à long terme et d'informer leur partenaire à cause de leurs inquiétudes au sujet du bébé par rapport aux femmes non enceintes.[37]

De même, aucune participante à l'étude n'a indiqué avoir eu plusieurs partenaires sexuels au cours de l'année précédant le diagnostic. Ce résultat n'est pas surprenant étant donné qu'une étude précédente a établi que seules 5% des femmes enceintes de Gaborone avaient eu deux ou davantage de partenaires au cours des 12 derniers mois.[4] Un autre article plus récent a montré, sur la base d'un sondage national, que 6% des femmes (non enceintes) en moyenne avaient eu deux ou davantage de partenaires sexuels au cours du dernier mois.[38] Bien que nous n'ayons pas pu analyser les expériences et les préférences de femmes aux multiples partenaires, c'est un élément important à prendre en compte lors de la mise en place de nouvelles stratégies d'annonce au partenaire. Des études récentes ont démontré l'importance d'adapter les services d'annonce au partenaire aux différents types de relations (par ex. couple stable, nouvelle relation, partenaire occasionnel, partenaire d'une seule fois) afin d'accroître leur efficacité et leur rentabilité.[39]

Enfin, il est important de noter que cette étude a été effectuée dans le cadre d'une étude plus large sur le dépistage des IST qui s'écartait du parcours de soins classique au Botswana, la prise en charge syndromique, dans laquelles les IST curables sont traitées en fonction des signes et des symptômes.[20] La prise en charge syndromique n'est pas systématique, elle passe à côté de nombreuses infections, ni spécifique, et traite donc potentiellement les femmes enceintes plus que nécessaire.[4] Par conséquent, quand les infections deréférence ne sont pas repérées, celles des partenaires non plus. En plus de cela, les femmes peuvent être encouragées à annonce une IST dont elles ne souffrent pas, ce qui les expose inutilement à des réactions négatives de la part de leurs partenaires, comme les VS.[2]

Au Botswana, les services d'annonce au partenaire pour le VIH sont semblables à ceux dédiés aux IST. Les femmes dont le dépistage du VIH est positif sont encouragées par les prestataires de soins à en informer leurs partenaires sexuels et les consignes incitent les prestataires de soins à proposer un accompagnement supplémentaire aux femmes réticentes ou inquiètes à cette idée.[40] De plus, les prestataires de soins sont autorisés à informer le partenaire d'une femme uniquement en sa présence et à sa demande.[40] Notre étude met en lumière les disparités entre hommes et femmes en termes d'accès aux services de santé, ce qui a été établi dans les études sur le VIH.[41] Non seulement cette disparité met davantage en danger la santé des hommes puisqu'ils sont moins susceptibles d'être dépistés et traités pour le VIH[41], mais en plus, cela oblige les femmes à protéger la santé de leurs partenaires pour ptotéger leur propre santé. Dans une telle situation, le renforcement de l'annonce aux partenaires et l'annonce ou la découverte de cas reposant sur les prestataires de soins recommandés pour les IST curables pourraient également faciliter l'annonce du VIH. De même,

étant donné que la gestion des IST et du VIH continuent à être intégrés aux soins prénatals au Botswana, il pourrait être possible d'harmoniser les services d'annonce au partenaire pour rationaliser le processus et accroître les taux d'annonce et d'orientation vers les services de soins pour des infections multiples.

CONCLUSIONS

En conclusion, le but de notre étude était de comprendre de manière plus approfondie les expériences et les préférences des femmes enceintes concernant l'annonce à leur partenaire d'une IST dans un envrionnement où la prévalence prénatale du VIH est élevée. Il est possible que le recours à des services de traitement des IST, du VIH et de soins prénatals ait contribué à la volonté de la plupart des femmes d'informer leurs partenaires. Cependant, des obstacles logistiques au traitement des partenaires subsistent. Pour faire augmenter les taux de partenaires contactés et traités avec succès, baisser le taux de réinfection au cours de la grossesse et enfin d'éviter les problèmes de santé de la mère et du nouveau-né attribuables à des IST contractées pendant la grossesse, des recherches supplémentaires sont nécessaires pour identifier les stratégies efficaces et adaptées au traitement des partenaires.

LISTE DES ABRÉVIATIONS

TAP	Traitement accéléré du partenaire
COREQ	Critères consolidés pour l'analyse d'une recherche qualitative (consolidated criteria for reporting qualitative research)
CT	*Chlamydia trachomatis*
VIH	Virus de l'immunodéficience humaine
VS	Violences sexuelles
NG	*Neisseria gonorrhoeae*
IST	Infections sexuellement transmissibles
TV	*Trichomonas vaginalis*
ONUSIDA	Programme commun des Nations Unies sur le VIH/SIDA
USD	Dollar américain
OMS	Organisation mondiale de la Santé

CONCERNANT CE SUPPLÉMENT

Cet article a été publié comme partie de l'ouvrage de *BMC Public Health*, Volume 19 Supplement 1, 2019: Effective Integration of Sexual Reproductive Health and HIV Prevention, Treatment, and Care Services across sub-Saharan Africa: Where is the evidence for program implementation? Le supplément a été publié dans le cadre d'une collaboration entre *Reproductive Health* et *BMC Public Health.* L'intégralité du contenu, avec les versions en français, en portugais et en anglais, est disponible en ligne : https://bmcpublichealth.biomedcentral.com/articles/supplements/volume-19-supplement-1 et https://reproductive-health-journal.biomedcentral.com/articles/supplements/volume-16-supplement-1

DÉCLARATIONS

Approbation éthique et accord de participation

Les commissions de révision de l'université du Botswana, du ministère de la Santé du Botswana, du Health Research Development Committee et du Princess Marina Hospital ont validé le protocole de cette étude. L'université de Californie à Los Angeles a validé les analyses utilisant des données anonymisées. Toutes les participantes à l'étude ont donné leur consentement par écrit avant d'être enrôlées. Toutes les données issues des entretiens ont été gardées confidentielles et stockées dans des lieux sécurisés. Au cours de la transcription, les éléments permettant d'identifier les personnes ont été supprimés et les documents ont été dotés d'un numéro d'identification pour l'étude. Une fois la transcription réalisée, les fichiers audio ont été détruits.

Accord de publication

Non applicable

Disponibilité des données et matériels

Non applicable

Conflits d'intérêts

Les auteurs déclarent ne pas avoir de conflits d'intérêts.

Financement

Le supplément de la revue est rendu possible grâce au soutien généreux du peuple américain via la United States Agency for International Development (USAID) en partenariat avec le Fonds des Nations unies pour la population (FNUAP) et le Programme commun des Nations Unies sur le VIH/SIDA (ONUSIDA).

Les opinions exprimées dans la présente publication sont celles des auteurs et ne reflètent pas nécessairement les politiques officielles de l'USAID, du FNUAP ou de l'ONUSIDA, la mention des dénominations de ministères ou d'organismes n'implique pas non plus l'aval du gouvernement américain, du FNUAP ou de l'ONUSIDA.

Contributions des auteurs

AW, OAO, DRM, CMorroni et JK ont participé à la conception et à la création de l'étude. NM a réalisé la collecte des données. AW, CM et OAO ont effectué l'analyse et interprété les résultats originaux. Tous les auteurs ont rédigé ou relu et approuvé le manuscrit final.

Remerciements

Les auteurs remercient l'équipe soignante de la maternité du Princess Marina Hospital pour son soutien et sa contribution à cette étude. Adriane Wynn a été soutenue par une bourse GloCal (2D43TW009343-06), un programme de formation T32 financé par le NIDA (T32DA023356) ainsi que par le Klausner Research and Training Fund. Ogechukwu Agatha Offorjebe a été soutenue par le David Geffen School of Medicine's Dean's Office et l'UCLA Center for World Health. Elles remercient les patientes pour leur participation.

RÉFÉRENCES

1. **Global Strategy for the Prevention and Control of Sexually Transmitted Infections: 2006-2015** [https://www.who.int/reproductivehealth/publications/rtis/9789241563475/en/]. Access date: September 3, 2017.
2. Alam N, Chamot E, Vermund S, al. e: **Partner notification for sexually transmitted infections in developing countries: a systematic review.** *BMC Public Health* 2010, **10**(19).
3. Althaus CL, Turner KM, Mercer CH, Auguste P, Roberts TE, Bell G, Herzog SA, Cassell JA, Edmunds WJ, White PJ *et al*: **Effectiveness and cost-effectiveness of traditional and new partner notification technologies for curable sexually transmitted infections: observational study, systematic reviews and mathematical modelling**. *Health technology assessment (Winchester, England)* 2014, **18**(2):1-100, vii-viii.
4. Romoren M, Sundby J, Velauthapillai M, Rahman M, Klouman E, Hjortdahl P: **Chlamydia and gonorrhoea in pregnant Batswana women: time to discard the syndromic approach?** *BMC Infect Dis* 2007, **7**:27.
5. Romoren M, Velauthapillai M, Rahman M, Sundby J, Klouman E, Hjortdahl P: **Trichomoniasis and bacterial vaginosis in pregnancy: inadequately managed with the syndromic approach**. *Bull World Health Organ* 2007, **85**(4):297-304.
6. Moodley D, Moodley P, Sebitloane M, Soowamber D, McNaughton-Reyes HL, Groves AK, Maman S: **High Prevalence and Incidence of Asymptomatic Sexually Transmitted Infections During Pregnancy and Postdelivery in KwaZulu Natal, South Africa**. *Sexually Transmitted Diseases* 2015, **42**(1):43-47.

7. Mcbride K GR, Fortenberry JD.: **Formative design and evaluation of patient-delivered partner therapy informational materials and packaging.** *Sex Transm Infect* 2009, **85**(2):150-155.
8. Gencay M, Koskiniemi M, Ammala P, Fellman V, Narvanen A, Wahlstrom T, Vaheri A, Puolakkainen M: **Chlamydia trachomatis seropositivity is associated both with stillbirth and preterm delivery**. *APMIS* 2000, **108**(9):584-588.
9. Rours GI DL, Moll HA, Arends LR, de Groot R, Jaddoe VW, Hofman A, Steegers EA, Mackenbach, JP OA, Willemse HF, van der Zwaan EA, Verkooijen RP, Verbrugh HA.: **Chlamydia trachomatis infection during pregnancy associated with preterm delivery: a population-based prospective cohort study. European journal of epidemiology.** *European Journal of Epidemiology* 2011, **26**(6):493-502.
10. Liu B RC, Clarke M, Jorm L, Hunt J, Ward J.: **Chlamydia and gonorrhoea infections and the risk of adverse obstetric outcomes: a retrospective cohort study.** *Sexually Transmitted Infections* 2013, **89**(8):672-678.
11. Cotch MF, Pastorek JG, 2nd, Nugent RP, Hillier SL, Gibbs RS, Martin DH, Eschenbach DA, Edelman R, Carey JC, Regan JA *et al*: **Trichomonas vaginalis associated with low birth weight and preterm delivery. The Vaginal Infections and Prematurity Study Group**. *Sex Transm Dis* 1997, **24**(6):353-360.
12. Fawzi W, Msamanga G, Renjifo B, Spiegelman D, Urassa E, Hashemi L, Antelman G, Essex M, Hunter D: **Predictors of intrauterine and intrapartum transmission of HIV-1 among Tanzanian women**. *AIDS* 2001, **15**(9):1157-1165.
13. Grosskurth H GR, Hayes R, Mabey D, Wawer M,: **Control of sexually transmitted diseases for HIV-1 prevention: Understanding the implications of the Mwanza and Rakai trial.** *Lancet* 2001, **355**(1981-1987).
14. World Health Organization: **Global Strategy for the Prevention and Control of Sexually Transmitted Infections: 2006-2015** *Accessed from: http://www.who.int/hiv/pub/toolkits/stis_strategy[1]en.pdf* 2007. Access date: January 8, 2019.
15. Gable L, Gostin L, Hodge J, Gamharter K, Van Puymbroeck R: **Legal aspects of HIV/AIDS: a guide for policy and law reform.** *Washington: The World Bank* 2007.
16. Alam N CE, Vermund S, et al.: **Partner notification for sexually transmitted infections in developing countries: a systematic review.** *BMC Public Health* 2010, **10**(19).
17. Ogechukwu A WA, Moshashane N, Sickboy O, Duque S, Ramogola-Masire D, Klausner JD, Morroni C.: **Partner Notificaiton and Treatment for Sexually Transmitted Infections Among Pregnant Women in Gaborone, Botswana.** *International Journal of STD & AIDS* 2016, **In submission**.
18. Statistics Botswana NACA, and Ministry of Health,: **Botswana AIDS Impact Survey 2013 (BAIS IV 2013)**. Accessed from: https://www.google.com/url?sa=t&rct=j&q=&esrc=s&source=web&cd=1&ved=2ahUKEwjq_a6J7t_fAhXLw1QKHSdMAswQFjAAegQIChAC&url=http%3A%2F%2Fwww.statsbots.org.bw%2Fsites%2Fdefault%2Ffiles%2Fpublications%2FBOTSWANA%2520AIDS%2520IMPACT%2520SURVEY%2520IV%25202013.pdf&usg=AOvVaw1Preoo1zbW8ACAbeNu8XLU. Access date: 8 January, 2019.

19. United Nations Development Programme: **HIV/ AIDS Overview: Botswana**. [http://www.bw.undp.org/content/botswana/en/home/ourwork/hiv_aids/overview.html] Access date: January 8, 2019.
20. Botswana Ministry of Health: **Management of Sexually Transmitted Infections. Reference Manual for Health Workers**. 2012.
21. Wynn A R-MD, Gaolebale P, Doherty K, Moshashane N, Sickboy O, Duque S, Williams L, Klausner JD, Morroni C.: **Prevalence and Correlates of Sexually Transmitted Infections Among Pregnant Women in Gaborone, Botswana, 2016**. *Sexually Transmitted Diseases* 2016, **In submission**.
22. Emerson R, Fretz R, Shaw L: **Writing Ethnographic Fieldnotes**. *Chicago: University of Chicago Press* 1995.
23. Tong A, Sainsbury P, Craig J: **Consolidated criteria for reporting qualitative research (COREQ): a 32-item checklist for interviews and focus groups**. *International journal for quality in health care : journal of the International Society for Quality in Health Care* 2007, **19**(6):349-357.
24. Moyo W, Chirenje ZM, Mandel J, Schwarcz SK, Klausner J, Rutherford G, McFarland W: **Impact of a Single Session of Counseling on Partner Referral for Sexually Transmitted Disease Treatment, Harare, Zimbabwe**. *AIDS and Behavior* 2002, **6**(3):237-243.
25. Harrison A, Lurie M, Wilkinson N: **Exploring partner communication and patterns of sexual networking: qualitative research to improve management of sexually transmitted diseases**. *Health Transit Rev* 1997, **7 Suppl 3**:103-107.
26. Zungu L, Salawu A, O'gunbanjo G: **Reported intimate partner violence amongst women attending a public hospital in Botswana**. *African Journal of Primary Health Care & Family Medicine* 2010, **2**(1).
27. Botswana Women's Affairs Department: **Gender Based Violence Indicators Study Botswana.** *Accessed from:* www.gov.bw/globalassets/mlha/gender-affairs/final-gbv-indicators-study-pamphlet---botswana.pdf. Access date: January 8, 2019.
28. Tafuma TA, Ntwayagae BC, Moalafhi CK, Bolebantswe JM: **Patient-initiated sexual partner notification in Botswana and time taken for sexual contacts to report for treatment**. *South African Medical Journal* 2013, **104**(1):42.
29. Centers for Disease Control and Prevention: **Expedited Partner Therapy**. *Accessed from: https://www.cdc.gov/std/ept/*. Access date: January 8, 2019.
30. Ferreira A, Young T, Mathews C, Zunza M, Low N: **Strategies for partner notification for sexually transmitted infections, including HIV**. *Cochrane Database Syst Rev* 2013(10):Cd002843.
31. Golden MR, Whittington WL, Handsfield HH, Hughes JP, Stamm WE, Hogben M, Clark A, Malinski C, Helmers JR, Thomas KK *et al*: **Effect of expedited treatment of sex partners on recurrent or persistent gonorrhea or chlamydial infection**. *N Engl J Med* 2005, **352**(7):676-685.
32. Estcourt C, Sutcliffe L, Cassell J, Mercer CH, Copas A, James L, Low N, Horner P, Clarke M, Symonds M *et al*: **Can we improve partner notification rates through expedited partner therapy in the UK? Findings from an exploratory trial of Accelerated Partner Therapy (APT)**. *Sex Transm Infect* 2012, **88**(1):21-26.

33. Quinn TC, Gaydos C, Shepherd M, Bobo L, Hook EW, 3rd, Viscidi R, Rompalo A: **Epidemiologic and microbiologic correlates of Chlamydia trachomatis infection in sexual partnerships**. *Jama* 1996, **276**(21):1737-1742.
34. Lycke E, Lowhagen GB, Hallhagen G, Johannisson G, Ramstedt K: **The risk of transmission of genital Chlamydia trachomatis infection is less than that of genital Neisseria gonorrhoeae infection**. *Sex Transm Dis* 1980, **7**(1):6-10.
35. Pellowski J, Mathews C, Kalichman MO, Dewing S, Lurie MN, Kalichman SC: **Advancing Partner Notification Through Electronic Communication Technology: A Review of Acceptability and Utilization Research**. *J Health Commun* 2016, **21**(6):629-637.
36. Botswana Central Statistical Office and UNICEF: **2007 Botswana Family Health Survey IV Report.** *Gaborone* 2009.
37. Garcia PJ, Williams E, Carcamo CP, Chiappe M, Holmes KK, Peeling RW, Mabey DM: **Partner Notification Among Peruvian Pregnant Women With Syphilis**. *Sex Transm Dis* 2015, **42**(8):457-462.
38. Ho-Foster A, Laetsang D, Masisi M, Anderson M, Tlhoiwe D, Cockcroft A, Andersson N: **Gender-specific patterns of multiple concurrent sexual partnerships: a national cross sectional survey in Botswana**. *AIDS Care* 2010, **22**(8):1006-1011.
39. Estcourt C: **Going beyond "regular and casual": developing a new sex partner classification to enable prioritisation of resources and tailoring of partner notification.** *Presentation at the IUSTI World & European Conference* 2018, **June 2018.**(Dublin, Ireland.).
40. Botswana Ministry of Health: **National Guidelines: HIV Testing and Counselling.** 2009.
41. Baker P, Dworkin SL, Tong S, Banks I, Shand T, Yamey G: **The men's health gap: men must be included in the global health equity agenda**. *Bull World Health Organ* 2014, **92**(8):618-620

Tableau 1. Caractéristiques des participantes à l'étude sur l'annonce au partenaire d'une infection sexuellement transmissible réalisée au Princess Marina Hospital de Gaborone au Botswana.

	**Échantillon étudié, N (%)**
Âge en années, moyenne (étendue)	29 (21-35)
Non mariées	15 (100 %)
Niveau d'études	
Collège ou moins	6 (40 %)
Lycée	3 (20 %)
Études supérieures	6 (40 %)
Séropositivité	6 (40 %)
Symptômes de l'IST mentionnés au moment du dépistage de l'IST*	7 (47 %)
Écoulement vaginal	4 (57 %)
Miction douloureuse	2 (29 %)
Douleurs au bas-ventre	3 (43 %)

Annonce du diagnostic d'IST au partenaire	13 (87 %)
Partenaire traité	
Oui	7 (47 %)
Non (y compris ceux qui n'ont pas été informés)	4 (27 %)
Incertain	4 (27 %)
IST guérie lors du suivi de la patiente de référence	12 (80 %)

Note : *Certaines femmes ont mentionné plusieurs symptômes. Les dénominateurs pour les écoulements vaginaux, la miction douloureuse et les douleurs au bas-ventre sont le nombre de femmes ayant au moins un symptôme. Le total des pourcentages peut ne pas être égal à 100 à cause de l'arrondi.

Tableau 2. Caractéristiques des femmes incluses dans l'échantillon d'entretiens qualitatifs

N° de la participante	IST	Séropositivité pour le VIH	Statut du partenaire	Partenaire informé	Partenaire traité	IST guérie**
1	TV	Séropositive	Pas de partenaire	Non	Non	Oui
2	TV	Séronégative	Nouveau partenaire	Non	Non	Oui
3	CT	Séropositive	Père du bébé	Oui	Non	Oui
4	CT	Séropositive	Pas de partenaire	Oui	Non	Oui
5	NG	Séropositive	Père du bébé	Oui	Incertain	Oui
6	CT	Séronégative	Père du bébé	Oui	Incertain	Non
7	TV	Séronégative	Père du bébé	Oui	Incertain	Oui
8	CT	Séronégative	Pas de partenaire	Oui	Incertain	Oui
9	CT	Séronégative	Père du bébé	Oui	Oui	Oui
10	CT	Séronégative	Père du bébé	Oui	Oui	Non
11	CT	Séronégative	Père du bébé	Oui	Oui	Non
12	CT	Séropositive	Père du bébé	Oui	Oui	Oui
13	TV	Séronégative	Père du bébé	Oui	Oui	Oui
14	TV	Séronégative	Père du bébé	Oui	Oui	Oui
15	CT	Séropositive	Pas de partenaire	Oui	Oui	Oui

Note : *Statut du partenaire au moment de l'entretien. **Les participantes ont été dépistées à nouveau au bout de quatre semaines pour vérifier la guérison.
