# Supplementary material for: Using partner notification to address curable sexually transmitted infections in a high HIV prevalence context: a qualitative study about partner notification in Botswana
Source: BMC Public Health. 2019 May 29;19(Suppl 1):606. doi: 10.1186/s12889-019-6813-2 (PMC6538557; doi:10.1186/s12889-019-6813-2)
Supplement: Supplementary file 2 — Translation of the abstract of this article into Portuguese. (PDF 376 kb) [file 12889_2019_6813_MOESM2_ESM.pdf]

BMC Public Health 2019, 19 (Suppl 1) [translated from the original publication]

Utilizar a notificação de parceiros para lidar com infeções sexualmente transmissíveis curáveis num contexto de elevada prevalência do VIH: Um estudo qualitativo sobre a notificação de parceiros no Botsuana

Adriane Wynn[1,2*], Corrina Moucheraud[3], Neo Moshashane[4], Ogechukwu Agatha Offorjebe[5,6], Doreen Ramogola-Masire[7], Jeffrey D Klausner[8], Chelsea Morroni[9, 10, 11, 12, 13]

[*1]GloCal, University of California Global Health Institute, 550 16th Street, 3rd Floor, San Francisco, CA 94158, USA,
[2] Division of Infectious Diseases & Global Public Health, Department of Medicine, University of California, San Diego, 9500 Gilman Drive, La Jolla, CA 92093, USA, Email : awynn@ucsd.edu ,
[3]Department of Health Policy and Management, University of California, Los Angeles, 31-269 CHS Box 951772, Los Angeles, CA 90095, USA, Email : cmoucheraud@g.ucla.edu,
[4] Botswana-UPenn Partnership, UB Main Campus, Gaborone, Botswana, Email : Moshashanen@bup.org.bw
[5]David Geffen School of Medicine, University of California, Los Angeles, 10833 Le Conte Avenue, Los Angeles, CA 90095, USA, Email : OOfforjebe@mednet.ucla.edu
[6]Charles R. Drew University of Medicine and Science, 1731 E 120th St, Los Angeles, CA 90059
[7]Faculty of Medicine, University of Botswana, Gaborone, Botswana, Email : doreen.masire@gmail.com
[8] David Geffen School of Medicine, University of California, Los Angeles, 10833 LE Conte Avenue, Los Angeles, CA 90095, USA, Email : JDKlausner@mednet.ucla.edu
[9] Botswana-UPenn Partnership, UB Main Campus, Gaborone, Botswana
[10] Liverpool School of Tropical Medicine, UK
[11] Department of Medicine, University of Botswana, Gaborone, Botswana
[12] Wits Reproductive Health and HIV Institute, University of Witwatersrand, Johannesburg, South Africa
[13] Women's Health Research Unit, University of Cape Town, South Africa, E-mail : chelseaamorroni@gmail.com

***Autor correspondente: Adriane Wynn** awynn@ucsd.edu

Resumo

Introdução: A notificação de parceiros é um componente essencial da gestão de infeções sexualmente transmissíveis (IST). O processo implica identificar parceiros sexuais expostos, notificar os mesmos sobre a sua exposição a uma IST curável, bem como oferecer aconselhamento e tratamento para a IST como parte da gestão sindrómica ou após os resultados de um teste de despistagem de IST. Quando eficazmente aplicados, os serviços de notificação de parceiros podem impedir que o doente inicial seja infetado novamente com uma IST curável a partir de um parceiro não tratado, reduzir o impacto das IST curáveis na comunidade e prevenir consequências negativas para a saúde do doente inicial e do respetivo parceiro sexual.

Contudo, as taxas de notificação de parceiros e de tratamento são frequentemente baixas. Este estudo procura explorar as experiências e preferências relacionadas com a notificação de parceiros e o tratamento de IST curáveis entre mulheres grávidas a receber cuidados de saúde numa clínica de cuidados pré-natais com testes de despistagem de VIH e IST curáveis integrados. Os resultados visam informar os esforços de melhorar as taxas de notificação de parceiros e de tratamento na África do Sul.

Métodos: Foram realizadas entrevistas qualitativas entre mulheres diagnosticadas com infeções por *Chlamydia trachomatis* (CT)*, Neisseria gonorrhoeae* (NG) e/ou *Trichomonas vaginalis* (TV) que procuravam cuidados pré-natais em Gaborone no Botsuana. As entrevistas

semiestruturadas foram utilizadas para saber que conhecimentos estas mulheres tinham relativamente às IST, bem como as suas experiências e preferências no âmbito da notificação de parceiros.

Resultados: 15 mulheres aceitaram participar no estudo. Antes da realização do teste, a maioria das inquiridas não tinha nenhum tipo de conhecimentos acerca das infeções por CT, NG ou TV. 13 das 15 participantes tinham informado os parceiros sobre o diagnóstico de IST. A maior parte dos parceiros notificados recebeu algum tipo de tratamento, mas o tratamento dos parceiros foi muitas vezes realizado tardiamente. A maioria das mulheres manifestou a sua preferência em acompanhar os parceiros até à clínica para serem tratados. As experiências e preferências não diferiram por estado da infeção do VIH.

Conclusões: A integração de serviços de IST, VIH e cuidados pré-natais podem ter contribuído para uma maior predisposição das mulheres notificarem os parceiros. Contudo, continuam a existir obstáculos logísticos ao tratamento dos parceiros. É necessária mais investigação para identificar estratégias eficazes e adequadas para desenvolver os serviços de notificação de parceiros a fim de melhorar as taxas de informação e tratamento de parceiros com êxito, reduzir as taxas de reinfeções de IST durante a gravidez e, em última análise, reduzir resultados negativos para as mães e os filhos atribuíveis às IST pré-natais.

Palavras-chave: notificação de parceiros, tratamento, infeções sexualmente transmissíveis, mulheres grávidas, *Chlamydia trachomatis, Neisseria gonorrhoeae, Trichomonas vaginalis*, África do Sul, VIH

Sobre este suplemento

Este resumo foi publicado como parte da revista científica *BMC Public Health*, Volume 19, Suplemento 1, 2019: Integração Eficaz dos Serviços de Saúde Sexual e Reprodutiva e de Prevenção, Cuidados e Tratamento do VIH na África Subsariana: Onde estão as provas da implementação do programa?
O suplemento foi publicado como uma colaboração entre as revistas científicas *Reproductive Health* e *BMC Public Health.* O conteúdo integral, incluindo as versões em francês, português e inglês, estão disponíveis online:
https://bmcpublichealth.biomedcentral.com/articles/supplements/volume-19-supplement-1
e
https://reproductive-health-journal.biomedcentral.com/articles/supplements/volume-16-supplement-1
